# Processing Effects on Protein–Polysaccharide Biointerfaces in Omega-3 Microencapsulation: Linking Structure with Oxidative Stability and Gastrointestinal Functionality

**DOI:** 10.3390/molecules31193408

**Published:** 2026-09-24

**Authors:** Marta Siol, Bartłomiej Zieniuk, Joanna Bryś, Ewa Domian, Diana Mańko-Jurkowska

**Affiliations:** 1 Department of Chemistry, Institute of Food Sciences, Warsaw University of Life Sciences, Nowoursynowska St. 159c, 02-787 Warsaw, Poland; marta_siol@sggw.edu.pl (M.S.); joanna_brys@sggw.edu.pl (J.B.); diana_manko-jurkowska@sggw.edu.pl (D.M.-J.); 2 Department of Food Engineering, Institute of Food Sciences, Warsaw University of Life Sciences, Nowoursynowska St. 159c, 02-787 Warsaw, Poland; ewa_domian@sggw.edu.pl

**Keywords:** protein–polysaccharide interactions, omega-3 fatty acids, food processing, microencapsulation, bioaccessibility, structure–function relationships

## Abstract

Omega-3 polyunsaturated fatty acids (PUFAs) are widely recognized for their nutritional and health benefits, yet their high susceptibility to oxidative degradation remains a major challenge in the development of stable food-grade delivery systems. Protein–polysaccharide-based microencapsulation provides an effective strategy for protecting omega-3-rich oils while modulating their release and accessibility during gastrointestinal digestion. However, the relationships among processing conditions, wall-material composition, resulting structural organization, and functional performance remain insufficiently integrated, limiting the establishment of general design principles for omega-3 delivery systems. This review critically examines these relationships from a processing–structure–function perspective, with particular attention to molecular interactions, interfacial organization, matrix architecture, drying and coacervation processes, oxygen transport, lipid oxidation, and gastrointestinal transformations. The reviewed evidence indicates that encapsulation efficiency alone is insufficient to predict functional performance. Instead, oxidative stability arises from the combined effects of interfacial organization, surface-oil distribution, matrix continuity, and oxygen transport, while gastrointestinal functionality depends on structural transformations that regulate lipid release, bile-salt-mediated interfacial remodeling, lipase accessibility, and bioaccessibility. Importantly, increasing interfacial or matrix resistance can improve oxidative or gastric protection but may also restrict intestinal lipid digestion, revealing a structure-dependent trade-off between protection and digestive accessibility. Effective omega-3 delivery systems therefore require structures that provide sufficient protection during processing and storage while allowing controlled reorganization during gastrointestinal digestion. This processing–structure–function framework can support the rational selection of wall-material combinations and processing conditions for omega-3-fortified foods and functional ingredients.

## 1. Introduction

Omega-3 polyunsaturated fatty acids (n-3 PUFAs), including α-linolenic acid (ALA), eicosapentaenoic acid (EPA), and docosahexaenoic acid (DHA), are among the most extensively investigated bioactive lipids because of their well-established nutritional and health benefits. Despite their recognized physiological importance, the successful incorporation of omega-3-rich oils into food systems remains technologically challenging owing to their exceptional susceptibility to oxidative degradation. Oxidation of highly unsaturated lipids leads to the formation of hydroperoxides and secondary oxidation products, resulting in deterioration of nutritional quality, development of undesirable sensory attributes, and reduced shelf life of fortified foods. These processes are governed not only by environmental factors but also by the physicochemical organization of the lipid phase, particularly the characteristics of the oil–water interface and the distribution of pro-oxidants within dispersed systems [1,2].

Beyond oxidative stability, the nutritional effectiveness of omega-3 lipids depends on their gastrointestinal fate. Lipid digestion, release, and bioaccessibility are strongly influenced by the structural characteristics of the delivery system, especially interfacial organization and the accessibility of lipid droplets to digestive enzymes [3,4]. Consequently, delivery systems designed to maximize oxidative protection do not necessarily provide optimal gastrointestinal performance. In many cases, structural modifications that improve oxidation resistance may simultaneously alter lipolysis, lipid release, and bioaccessibility, demonstrating that oxidative stability and delivery functionality should be considered interconnected rather than independent design objectives [5,6,7].

Microencapsulation has therefore emerged as one of the most effective approaches for protecting omega-3-rich oils and facilitating their incorporation into food products. However, growing evidence indicates that encapsulation performance is determined not simply by the encapsulation technique or wall material composition, but by the structural organization established throughout the system. The architecture of the interfacial layer, the spatial distribution of lipids within the encapsulating matrix, and the resulting transport properties collectively determine oxygen accessibility, oxidative stability, and digestive behaviour [1,6,8,9]. Among the numerous wall-material strategies investigated, protein–polysaccharide systems have attracted particular attention because they enable the rational engineering of encapsulation structures through electrostatic interactions, hydrogen bonding, hydrophobic associations, and complex coacervation [1,7,10,11]. These molecular interactions generate interfacial and matrix architectures with distinct barrier properties, thereby influencing oxygen diffusion, droplet stability, matrix integrity, and gastrointestinal performance [3,6]. Rather than acting as passive encapsulating materials, proteins and polysaccharides function as structural building blocks whose interactions ultimately determine the functional behaviour of omega-3 delivery systems.

Despite the rapid expansion of this research area, the current literature remains highly fragmented. As a consequence, it remains difficult to distinguish whether differences in oxidative stability arise primarily from wall-material composition, encapsulation technology, or the structural organization generated during processing. Published studies differ considerably with respect to omega-3 sources, wall-material combinations, encapsulation techniques, oxidation protocols, digestion models, and analytical methodologies, making direct comparison between studies difficult and limiting the establishment of general design principles [6,8]. Previous reviews on omega-3 microencapsulation have primarily focused on encapsulation technologies, wall-material selection, physicochemical characterization, and oxidative stability [1,10,12,13]. Some studies have extended this perspective to gastrointestinal digestion and bioavailability, particularly in complex-coacervate systems; however, these outcomes have generally been considered as downstream performance characteristics rather than being systematically linked to the structural organization established during processing [6]. More recent reviews of protein–polysaccharide delivery systems provide a deeper mechanistic understanding of biopolymer interactions, interfacial organization, and controlled release, but their scope encompasses diverse bioactive compounds and delivery architectures rather than focusing specifically on the challenges associated with omega-3 microencapsulation [7,11]. Consequently, comparatively less attention has been devoted to integrating processing conditions, protein–polysaccharide interactions, interfacial and matrix architecture, oxidative stability, and gastrointestinal functionality within a unified processing–structure–function framework specifically for omega-3 delivery systems. Nevertheless, the available evidence indicates that processing-induced changes in biopolymer interactions and structural organization can influence both the physicochemical stability and digestive behavior of encapsulated bioactives, supporting the need to consider these relationships within an integrated framework [6,7,11].

Therefore, this review provides a critical synthesis of recent advances in protein–polysaccharide-based omega-3 microencapsulation from a processing–structure–function perspective. Rather than reviewing encapsulation techniques or wall materials in isolation, it examines how processing conditions and wall-material composition govern molecular interactions, interfacial organization, and matrix architecture, and how these structural features subsequently determine oxidative stability and gastrointestinal performance. By integrating processing, molecular interactions, structural organization, and functional outcomes within a unified mechanistic framework, this review aims to support the rational design of next-generation food-grade omega-3 delivery systems and to identify the major challenges that must be addressed to optimize both oxidative protection and gastrointestinal functionality. From a practical perspective, this integrated approach can support the selection of wall-material combinations and processing conditions that provide sufficient oxidative protection during processing and storage while maintaining appropriate lipid release and bioaccessibility during digestion, which is particularly relevant to the development of omega-3-fortified foods and functional ingredients.

## 2. Bibliometric Analysis of Research Trends in Omega-3 Microencapsulation

The bibliometric mapping revealed a highly interconnected research landscape centered on three major dimensions: encapsulation technologies, wall-material design, and functional performance of omega-3 delivery systems (Figure 1). Among the most prominent concepts in the network were microencapsulation, encapsulation, omega-3 fatty acids, and oxidative stability, accompanied by terms related to processing technologies, including spray drying, complex coacervation, freeze-drying, and nanoemulsions. Protein- and polysaccharide-related terms, including chitosan, maltodextrin, pectin, casein, whey protein, and sodium alginate, were distributed across different areas of the network, indicating that wall-material selection represents a recurring theme across multiple technological approaches.

A particularly prominent research direction concerns the relationship between encapsulation and oxidative protection. The close association of oxidative stability and lipid oxidation with terms related to microencapsulation, wall materials, and encapsulation efficiency reflects the established importance of oxidative protection as a major performance criterion for omega-3 delivery systems. In contrast, digestion-related concepts, including digestion, in vitro digestion, and bioavailability, occupy a less dominant and more dispersed position within the network. This difference suggests that gastrointestinal performance has been incorporated into omega-3 encapsulation research less consistently than oxidative stability, and remains less closely integrated with wall materials and structural considerations.

The network also reflects the coexistence of established and emerging encapsulation approaches. Conventional technologies such as spray drying, freeze-drying, and complex coacervation occur alongside nanoemulsions, nanoparticles, electrospraying, and electrospinning. This distribution indicates that the field is developing through both the optimization of established food-grade encapsulation technologies and the exploration of more advanced delivery architectures. The simultaneous occurrence of terms related to proteins, polysaccharides, emulsions, and biopolymers further suggests that material and interfacial design remain relevant across both technological directions.

Taken together, the bibliometric mapping reveals an important imbalance in the current research landscape. The stronger representation of oxidative-stability-related concepts compared with digestion- and bioavailability-related concepts suggests that oxidative protection remains more firmly established as a performance criterion in omega-3 encapsulation research, whereas gastrointestinal functionality is less consistently integrated with wall-material and structural considerations. Moreover, the dispersion of processing technologies, wall materials, and functional outcomes across the network reflects the heterogeneity of approaches used to investigate omega-3 delivery systems. These observations highlight the need for an integrative framework that connects processing conditions and protein–polysaccharide interactions to the resulting interfacial and matrix structures, and, subsequently, to both oxidative stability and gastrointestinal functionality. This processing–structure–function relationship, therefore, provides the conceptual framework for the following sections of this review.

## 3. Microencapsulation as a Strategy for Protection and Delivery of Omega-3 Fatty Acids

Microencapsulation encompasses a diverse group of technologies designed to improve the stability and functionality of omega-3-rich oils in food systems. Although these technologies differ considerably in their processing principles, they share a common objective: generating protective structures that control the physicochemical environment surrounding the lipid phase [6,8]. Consequently, encapsulation should be viewed not simply as a preservation technique but as a strategy for engineering delivery systems with specific interfacial architectures, matrix organizations, and transport properties.

The performance of encapsulated omega-3 systems is therefore determined not only by the encapsulation technique itself but also by the interactions between processing conditions, wall-material composition, and microstructural organization. Common wall materials include proteins such as whey protein, casein, and gelatin, as well as plant-derived proteins, and polysaccharide- and carbohydrate-based materials such as gum arabic, alginate, pectin, carrageenan, chitosan, and maltodextrin [14]. Proteins primarily contribute to emulsification and interfacial stabilization by adsorbing at the oil–water interface and forming protective films around lipid droplets, whereas polysaccharide- and carbohydrate-based materials can enhance matrix formation, viscosity, and barrier properties [14,15]. Their combination can further promote the formation of cohesive interfacial or matrix structures, thereby improving encapsulation efficiency and limiting the exposure of omega-3 lipids to pro-oxidative environmental factors [15,16]. These structural features collectively govern oxygen diffusion, lipid mobility, barrier integrity, and the accessibility of encapsulated lipids to digestive enzymes, ultimately influencing both oxidative stability and gastrointestinal performance [6,8]. Understanding how different encapsulation routes generate distinct structural organizations is therefore fundamental for the rational design of efficient omega-3 delivery systems.

### 3.1. Definition and Functional Rationale of Microencapsulation

Microencapsulation is broadly defined as the formation of a functional barrier between an active core and a surrounding wall material, coating, or matrix in order to reduce undesirable interactions between the encapsulated compound and its environment [17,18,19]. In the case of omega-3-rich oils, this approach converts highly oxidation-prone liquid lipids into structured ingredients with improved stability, handling, and applicability in food systems. Beyond simple physical entrapment, microencapsulation enables engineering of the local microenvironment surrounding lipid droplets. By controlling interfacial organization, matrix architecture, and oxygen permeability, encapsulation systems can modify both oxidative stability and gastrointestinal behaviour of omega-3-rich oils. In addition to protecting lipids against environmental stresses, microencapsulation improves formulation flexibility, masks undesirable sensory attributes, and, depending on particle architecture, may provide controlled lipid release during digestion [18,20,21].

Consequently, microencapsulation should be regarded as a structure-engineering strategy in which material selection, processing conditions, and microstructural organization collectively determine the functional performance of omega-3 delivery systems.

### 3.2. Main Degradation Pathways of Omega-3 Lipids in Encapsulated Systems and the Protective Role of Encapsulation

Although the high oxidative susceptibility of omega-3 fatty acids has been introduced in the previous section, oxidative deterioration within encapsulated systems is governed largely by microstructural rather than compositional factors. The extent of lipid oxidation depends not only on the fatty acid profile of the encapsulated oil but also on the accessibility of oxygen and pro-oxidants through the capsule architecture. Consequently, oxidative stability is strongly influenced by wall continuity, interfacial organization, particle porosity, and the spatial distribution of lipids within the encapsulating matrix. One of the major determinants of oxidative instability is surface oil, i.e., lipid located at or near the particle surface rather than fully entrapped within the encapsulating matrix. Because it is directly exposed to atmospheric oxygen, surface oil is the primary site of oxidation in spray-dried powders and related systems, oxidizing several-fold faster than oil retained within the particle interior, thereby accelerating the formation of primary and secondary oxidation products [22,23,24]. Consequently, minimizing surface oil is regarded as one of the principal objectives in the design of stable omega-3 delivery systems. Another critical determinant is oxygen diffusion through the wall matrix. Porous, cracked, or poorly consolidated particles facilitate oxygen penetration, particularly when defects generated during dehydration or freeze-drying compromise barrier integrity, thereby accelerating oxidation. Therefore, the protective capacity of microencapsulation depends not only on the wall material composition but also on the ability of the selected processing route to generate dense, continuous, and low-permeability structures.

In addition to the bulk wall matrix, the interfacial layer surrounding lipid droplets constitutes the first barrier against oxidation, with its composition, thickness, and integrity regulating oxygen diffusion, pro-oxidant transport, and droplet stability during processing and storage. Consequently, interfacial organization provides a direct mechanistic link between wall-material composition, particle architecture, and oxidative performance, emphasizing that oxidation resistance is determined by the entire hierarchical structure of the encapsulated system rather than by the wall material alone. The protective performance of encapsulated systems may also deteriorate during storage or gastrointestinal exposure. For example, Jia et al. [25] demonstrated that elevated temperatures and acidic conditions compromised the structural integrity of spray-dried fish oil microcapsules formulated with OSA-modified starch and maltodextrin, thereby accelerating lipid oxidation during storage and increasing oil release under simulated gastric conditions. During 30 days of storage, lipid oxidation was most pronounced at 60 °C and lowest at 4 °C, while after three months at 60 °C, the microcapsules exhibited severe structural disintegration and shell rupture, in contrast to the largely preserved morphology observed at 4 °C. The same system also showed strong pH-dependent release under simulated gastrointestinal conditions: after 120 min, core-material release reached 83.73 ± 1.04% in simulated gastric fluid (pH 1.0), compared with only 33.55 ± 0.65% in simulated intestinal fluid (pH 6.8) [25]. These findings illustrate that the barrier function of encapsulation evolves dynamically throughout storage and digestion. Likewise, destabilization of the precursor emulsion, for example, through thermal denaturation of protein-based wall materials or emulsifiers, promotes droplet aggregation, reduces oil retention, and compromises oxidative stability, emphasizing that emulsion stability before drying is a prerequisite for effective encapsulation.

Collectively, these observations demonstrate that encapsulation efficiency alone is an insufficient predictor of oxidative stability. Delivery systems exhibiting comparable oil retention may differ substantially due to variations in surface oil content, oxygen permeability, interfacial organization, and particle microstructure. Thus, oxidative stability should be regarded as an emergent property arising from the combined effects of wall material composition, processing-induced architecture, and structural integrity during storage and digestion, rather than as a direct consequence of encapsulation efficiency alone [21,23]. However, a recurring limitation of the available literature is that these structural determinants are rarely evaluated simultaneously, making it difficult to establish their relative contributions to oxidation. The major degradation factors affecting omega-3-rich oils, together with the principal encapsulation mechanisms used to improve their oxidative stability and gastrointestinal performance, are schematically summarized in Figure 2.

### 3.3. Encapsulation Mechanisms Relevant to Omega-3 Systems

Although numerous encapsulation technologies have been developed for omega-3-rich oils, the structural characteristics of the resulting delivery systems depend on both the preparation method and the wall materials applied [8,18]. The same encapsulation technology can generate different particle morphologies depending on the wall material composition and processing conditions, including pH, biopolymer concentration, and mixing conditions [8,9]. Consequently, encapsulation technologies should be regarded not simply as alternative processing routes but as strategies for engineering interfacial and matrix structures that determine oxygen transport, lipid mobility, and the accessibility of encapsulated lipids during gastrointestinal digestion [6,18]. The principal structural mechanisms include matrix-type encapsulation, shell-forming systems such as complex coacervates, crosslinked networks, and multilayer assemblies formed through sequential deposition of biopolymers. In matrix-type systems, such as spray-dried powders, emulsified lipid droplets are immobilized within a continuous biopolymer matrix, and oxidative protection is strongly influenced by matrix continuity, surface oil content, and transport properties [6,8,18]. In contrast, complex coacervation and multilayer deposition can generate more defined interfacial barriers around lipid droplets, whereas ionic or covalent crosslinking can increase network cohesion and resistance to structural disruption. Thus, the effectiveness of encapsulation depends not only on the nominal technique but also on the resulting interfacial organization, matrix density, and structural integrity.

Experimental findings demonstrate that these processing-induced structural differences translate into measurable changes in encapsulation performance. Semenoglou et al. [15] showed that introducing a two-step homogenization process, including high-pressure homogenization at 60 MPa for four cycles, reduced the mean droplet diameter of fish-oil emulsions from 2.15 µm to 272.1 nm and increased encapsulation efficiency by 15.9–29.7% compared with one-step homogenization. In the two-step system containing carboxymethyl cellulose (CMC) and maltodextrin, encapsulation efficiency reached 82.4%, while the K232 value after eight weeks of storage at 43% relative humidity was 3.7 ± 0.1, compared with 9.3 ± 0.2 for the formulation without additional polysaccharides. The same CMC-maltodextrin system achieved an omega-3 bioaccessibility of 29.6 ± 3.9%, compared with 15.5 ± 2.0% for non-encapsulated fish oil. These findings demonstrate that processing conditions and matrix composition jointly affect droplet organization, lipid entrapment, oxidative protection, and subsequent gastrointestinal accessibility.

Structural reinforcement may, however, involve a trade-off between oxidative protection and lipid accessibility during digestion. Chen et al. [26] investigated fish gelatin–sodium alginate coacervates subjected to intra-particle crosslinking at different genipin concentrations. During accelerated storage at 63 °C, the uncrosslinked microcapsules reached a peak peroxide value of 659 ± 3 mmol/kg oil, whereas crosslinking at a 1.0% genipin-to-gelatin mass ratio reduced the peak value to 442 ± 3 mmol/kg oil. The authors attributed the improved oxidative resistance to the formation of a more stable crosslinked structure. However, increasing structural reinforcement also reduced lipid accessibility during digestion: free fatty acid release after 120 min of intestinal digestion decreased from 39.2 ± 2.6% in the uncrosslinked system to 31.6 ± 1.1% at 1.0% genipin and further to 25.2 ± 2.8% at 4.0% genipin. Thus, increasing network cohesion can enhance resistance to oxidative deterioration while simultaneously restricting structural disruption and lipase accessibility during digestion.

Collectively, these findings demonstrate that encapsulation efficiency alone is insufficient to predict the functional performance of omega-3 delivery systems. Instead, performance emerges from the combined effects of interfacial organization, matrix continuity, crosslinking density, and the structure’s ability to transform during storage and gastrointestinal transit. Dense, continuous structures may restrict oxygen and pro-oxidant transport, thereby delaying lipid oxidation, but excessive interfacial reinforcement or matrix rigidity may also limit enzyme accessibility and delay lipolysis. Conversely, structures that preserve droplet dispersion or undergo controlled disassembly during digestion may enhance lipid accessibility and subsequent bioaccessibility [5,27]. Therefore, the optimal architecture of an omega-3 delivery system requires a balance between sufficient barrier integrity during processing and storage and appropriate structural responsiveness during gastrointestinal digestion.

### 3.4. Overview of Encapsulation Techniques

#### 3.4.1. Spray Drying (SD)

Spray drying is the most widely used microencapsulation technique for omega-3-rich oils because it offers continuous operation, relatively low production costs, and straightforward industrial scalability [22,28,29]. In this process, an oil-in-water emulsion is atomized into a heated chamber, where rapid evaporation of water immobilizes lipid droplets within a glassy biopolymer matrix, producing free-flowing powders suitable for food applications. Despite the use of elevated inlet temperatures, the short residence time generally limits the thermal degradation of encapsulated lipids, making spray drying applicable to fish-, algal-, and plant-derived omega-3 oils [22,30,31].

From a mechanistic perspective, the performance of spray drying depends less on the drying process itself than on the microstructure generated during particle formation. Rather than producing ideal core–shell capsules, spray drying immobilizes lipid droplets within a continuous matrix, making oxidative stability dependent on matrix continuity, oxygen permeability, particle morphology, and, in particular, surface oil content [22,32]. Because surface-localized oil remains directly exposed to atmospheric oxygen, minimizing its content and generating dense, low-permeability matrices are primary objectives in the design of spray-dried omega-3 delivery systems [22,24].

The quality of the final particles is largely determined by the characteristics of the precursor emulsion and the functionality of the wall material. Stable emulsions with small droplets promote homogeneous lipid distribution and reduce surface oil, whereas emulsion destabilization before drying favours droplet coalescence, heterogeneous particle formation, and reduced oxidative stability [22,29]. Within protein–polysaccharide systems, the balance between interfacial stabilization by proteins and matrix formation by polysaccharides is particularly important, as it governs barrier continuity during dehydration. Collectively, these studies indicate that optimizing wall-material composition is often more important than modifying drying conditions alone. For example, Özyurt et al. (2022) [33] compared different sodium caseinate-to-maltodextrin ratios in spray-dried fish oil microcapsules and found that the 1:2 formulation provided the greatest oxidative protection, with a peroxide value of 5.46 ± 1.00 mEq/kg and a TBA value of 0.27 ± 0.02 mg MA/kg. Encapsulation efficiency was comparable among the investigated formulations, indicating that differences in oxidative performance were primarily attributable to wall-material composition rather than to encapsulation efficiency alone. Similarly, Yu et al. [32] demonstrated that double-encapsulated fish-oil microcapsules with a stable two-shell structure achieved an encapsulation efficiency of 93.71% and released only 11.8% of the core material after 30 min in a 100 °C water bath. These findings indicate that reinforcing the core–shell architecture can improve the thermal resistance of encapsulated fish oil.

Overall, spray drying remains the industrial benchmark for omega-3 microencapsulation owing to its scalability and economic feasibility. However, its effectiveness is governed primarily by the microstructure generated during drying rather than by the process itself. Within protein–polysaccharide systems, oxidative stability depends on the formulation’s ability to minimize surface oil, reduce oxygen permeability, and preserve particle integrity during storage and gastrointestinal digestion. Despite its widespread use, spray-drying optimization still relies largely on empirical formulations rather than predictive structure–function relationships.

#### 3.4.2. Complex Coacervation (CC)

Complex coacervation is an effective encapsulation strategy for omega-3-rich oils when high oil loading and the formation of a well-defined interfacial barrier are required. The technique is based on associative phase separation between oppositely charged biopolymers, which assemble around dispersed oil droplets under controlled physicochemical conditions [1,6]. Unlike matrix-type encapsulation, complex coacervation produces a distinct interfacial shell whose formation and properties are strongly dependent on charge balance between the interacting polymers. Therefore, pH, biopolymer ratio, ionic strength, molecular characteristics of the wall materials, and subsequent crosslinking can determine coacervate yield, shell organization, encapsulation efficiency, oxidative protection, and lipid accessibility during digestion [6,34].

The importance of electrostatic balance was demonstrated quantitatively by Chen et al. [35] in OSA-modified starch-chitosan coacervates containing flaxseed oil. Increasing pH from 5.0 to 6.5 increased coacervation yield from 30.31 ± 1.30% to 48.77 ± 1.30%, while microcapsule size increased from 22.72 ± 6.15 µm at pH 5.5 to 35.17 ± 7.62 µm at pH 6.5. At pH 6.5, encapsulation efficiency reached 95.1 ± 0.57%, compared with 66.7% in the unadjusted control. Structural analyses revealed a compact three-dimensional viscoelastic network surrounding the oil droplets at the optimal pH. This structural reinforcement was accompanied by improved oxidative stability: the Rancimat induction period increased from 2.29 ± 0.05 h for free flaxseed oil to 2.81 ± 0.03 h for the pH 6.5 microcapsules, while the estimated shelf life at 25 °C increased from 9.94 to 18.82 ± 1.83 days. These findings provide a direct processing–structure–function relationship, demonstrating that pH-dependent charge regulation governs coacervate organization and consequently affects both oil entrapment and oxidative protection.

The protein-to-polysaccharide ratio represents another critical determinant of complex formation. Mu et al. [36] found that a chitosan to OSA-starch mass ratio of 1:3 at pH 6.0 produced a near-neutral ζ-potential (−0.77 ± 0.12 mV), the highest optical density (0.573 ± 0.019), and a maximum coacervate yield of 65.18 ± 3.04%. The study also demonstrated the importance of core loading: increasing algae-oil content from 5 to 25 wt% decreased encapsulation efficiency from 93.1 ± 1.2% to 42.8 ± 0.8%. Thus, efficient coacervation requires not only favorable electrostatic interactions between wall polymers but also an appropriate balance between the amount of interfacial material and the dispersed lipid phase.

Wall-material modification can further alter the interfacial properties of the resulting coacervates. Pham et al. [37] showed that modification of flaxseed protein isolate (FPI) with hydroxytyrosol prior to coacervation with flaxseed gum increased encapsulation efficiency from 87.8 ± 1.7% for unmodified FPI/FG to 95.4 ± 1.5%, while reducing surface oil from 2.8 ± 0.4% to 1.0 ± 0.3%. Surface protein coverage simultaneously increased from 36.9% to 49.2%, indicating enhanced protein accumulation at the particle surface. Interestingly, modification with flaxseed polyphenols produced stronger oxidative protection despite a lower encapsulation efficiency than the hydroxytyrosol-modified system, indicating that oxidative stability is determined not only by physical shell integrity but also by the chemical and antioxidant functionality of the wall materials. Therefore, encapsulation efficiency alone should not be considered a sufficient predictor of oxidative performance.

Structural characteristics of the coacervate also influence gastrointestinal lipid accessibility. Yang et al. [38] demonstrated that the source of gelatin in gelatin-sodium hexametaphosphate coacervates substantially affected lipid digestion. After simulated small-intestinal digestion, free fatty acid release reached 55.5% for porcine-skin gelatin-based systems, compared with 34.1% for fish gelatin and only 27.0% for bovine-skin gelatin. The authors associated this behavior with differences in molecular weight and gel strength, with stronger gelatin networks restricting pancreatic lipase accessibility to the encapsulated lipid. In the same study, the addition of sodium octenyl succinate starch as a freeze-drying aid prevented structural disruption during drying and resulted in encapsulation efficiencies above 95%, whereas the system without this protective component exhibited an oil-loading capacity of only 1.7%. These results demonstrate that both primary coacervate organization and structural preservation during subsequent processing determine the functional behavior of the final delivery system.

A similar structure-dependent release behavior was observed by Eratte et al. [39] for transglutaminase-crosslinked whey protein isolate-gum Arabic coacervates containing tuna oil. Oil release remained approximately 0.5% during the oral phase and 7.8–8.0% during simulated gastric digestion, but increased to 39.4–41.0% during the intestinal phase. This selective release behavior indicates that a sufficiently cohesive coacervate network can restrict premature lipid release under gastric conditions while remaining susceptible to enzymatic disruption in the intestine.

Collectively, these studies demonstrate that complex coacervation should be considered a structure-engineering process rather than simply a method for achieving high encapsulation efficiency. Optimal performance requires appropriate electrostatic balance during coacervate formation, sufficient interfacial coverage, and adequate structural integrity during subsequent drying and storage. However, increasing shell density or network strength may also restrict lipase accessibility during digestion. Therefore, the design of coacervate-based omega-3 delivery systems requires a balance between barrier integrity sufficient to limit oxygen exposure during processing and storage and structural responsiveness that enables lipid release and digestion under gastrointestinal conditions.

#### 3.4.3. Ionic Gelation and Related Extrusion-Based Approaches

Ionic gelation and related extrusion-based techniques represent mild encapsulation strategies particularly suited for omega-3-rich oils when thermal degradation must be minimized and gastrointestinal release controlled. In these systems, lipid droplets are dispersed in a hydrocolloid solution that is subsequently solidified by ionic crosslinking, most commonly via interactions between anionic polysaccharides, such as alginate or low-methoxyl pectin, and calcium ions [1,40]. The resulting three-dimensional hydrogel network entraps the lipid phase and acts as a diffusion barrier whose effectiveness depends primarily on matrix density, degree of crosslinking, and structural organization. Unlike thermal drying methods, ionic gelation is performed under mild conditions, making it well-suited for encapsulating highly oxidation-sensitive polyunsaturated lipids. The performance of ionically gelled systems is governed largely by the permeability and integrity of the hydrogel network rather than by the gelation process itself. Polymer composition, crosslinking conditions, capsule size, and lipid-droplet distribution collectively determine oxygen diffusion, water transport, and enzyme accessibility, thereby influencing oxidative stability and release behaviour [1,41]. An additional advantage of these systems is their pH-responsive behaviour: hydrogel matrices often remain relatively stable under gastric conditions but swell or disintegrate in the intestinal environment, enabling delayed lipid release and improved site-specific delivery [41,42]. Experimental evidence indicates that hydrogel matrix properties are the primary determinants of encapsulation performance. Menin et al. [41] reported that pectin-based flaxseed oil microcapsules exhibited high encapsulation efficiency and improved oxidative stability, while smaller capsules were more susceptible to oxidation because of their higher surface-area-to-volume ratio. Similarly, alginate-salep systems developed by Wissam and Samer [40] achieved encapsulation efficiencies exceeding 90% and exhibited limited gastric but extensive intestinal oil release, confirming the importance of matrix composition in regulating gastrointestinal functionality.

Recent developments indicate that ionic gelation can be combined with complementary encapsulation technologies to further improve capsule performance. For example, combining in situ calcium crosslinking with spray drying (CLAMs) improved the storage stability of EPA- and DHA-rich oils, particularly when OSA-modified starch was incorporated into the wall matrix, resulting in lower surface oil and improved oxidative stability [29]. Similarly, integrating ionotropic gelation with electrospraying produced multicore fish-oil capsules with high encapsulation efficiency and reduced peroxide formation, demonstrating that improved emulsion stability and matrix organization can further strengthen barrier properties [43]. These hybrid approaches illustrate that combining different structuring mechanisms may simultaneously enhance oxidative protection, storage stability, and controlled gastrointestinal release.

Overall, ionic gelation offers a versatile platform for designing diffusion-controlled delivery systems under mild processing conditions. Rather than the gelation process itself, hydrogel permeability, integrity, and responsiveness ultimately determine oxygen transport, oxidative stability, and lipid release during gastrointestinal digestion. The main limitation of these systems remains the trade-off between mechanical stability and controlled release, which is rarely optimized systematically across studies.

#### 3.4.4. Freeze-Drying (FD) and Freeze-Based Drying Approaches

Freeze-drying is widely used for encapsulating omega-3-rich oils because it removes water by sublimation under reduced pressure, thereby avoiding the high temperatures associated with spray drying [29,44]. This mild process improves the preservation of thermolabile compounds while reducing heat-induced lipid degradation. However, freezing and subsequent ice sublimation generate porous structures that facilitate oxygen diffusion and influence barrier performance.

Several comparative studies indicate that freeze-based drying generally provides better oxidative preservation during storage than conventional spray drying, although often at the expense of encapsulation efficiency and powder structure. For example, spray-freeze-dried carotenoid-enriched flaxseed oil exhibited substantially lower peroxide values during storage than spray-dried powders [45]. Similarly, Yang et al. [46] reported that freeze-based drying methods better preserved fish oil during storage, whereas spray drying achieved the highest encapsulation efficiency. These findings suggest that the lower thermal load of freeze-based processes may benefit highly oxidation-sensitive EPA- and DHA-rich oils. The main limitation of freeze-drying is the formation of highly porous matrices after ice-crystal sublimation, which facilitates oxygen diffusion and reduces barrier performance [29,47,48]. Accordingly, spray-dried particles generally exhibit higher encapsulation efficiency and more compact morphologies, whereas freeze-dried powders are typically more porous and structurally irregular [47,48]. These observations indicate that oxidative stability depends not only on drying temperature but also on the resulting microstructure. Accordingly, the performance of freeze-dried systems is strongly influenced by emulsion formulation and wall-material design. Fioramonti et al. [49] demonstrated that maltodextrin increased encapsulation efficiency in whey protein-alginate multilayer systems, whereas Wang et al. [50] reported improved oxidative stability of freeze-dried flaxseed oil powders formulated with lentil protein isolate-carrageenan complexes, highlighting the importance of stable protein–polysaccharide interfaces before freezing.

Overall, freeze-based drying should not be regarded as inherently superior to spray drying. Instead, the two approaches offer complementary advantages: spray drying is preferred for industrial scalability and high encapsulation efficiency, whereas freeze-based drying is better suited for preserving highly labile lipids and other thermosensitive compounds. These findings indicate that comparisons based solely on oxidation markers may be misleading, as the apparent superiority of freeze-drying often reflects reduced thermal stress, whereas long-term stability is governed by matrix architecture.

#### 3.4.5. Spray Chilling and Selected Emerging Approaches

Spray chilling represents an alternative encapsulation strategy in which the active oil is dispersed in a molten lipid carrier and atomized into a cooled chamber, where the wall material solidifies by crystallization rather than dehydration [51]. Unlike spray drying, this process avoids extensive water removal and high drying temperatures, making it attractive for heat-sensitive lipophilic compounds. The protective performance of spray-chilled particles is determined primarily by the crystallization behaviour, polymorphism, and compactness of the lipid matrix, which collectively influence oxygen permeability and release characteristics.

Experimental studies indicate that spray chilling provides effective oxidative protection while supporting gastrointestinal delivery. Santos et al. [51] demonstrated improved carotenoid retention during storage and higher bioaccessibility than bulk oil, whereas combining spray drying with spray chilling further reduced degradation, highlighting the potential of hybrid encapsulation strategies. Similarly, Xiao et al. [52] developed a self-emulsifying agarose derivative that served simultaneously as an emulsifier and a wall material, illustrating the growing interest in simplified formulations that integrate interfacial stabilization with matrix formation. Beyond spray chilling, increasing attention has also been devoted to electrohydrodynamic processing, multilayer emulsions, liposomes, nanoemulsions, solid lipid nanoparticles, and nanostructured lipid carriers. These systems are designed not only to protect omega-3 lipids but also to improve their gastrointestinal fate by controlling interfacial organization and lipid digestion [53,54]. Electrohydrodynamic techniques have been reported to achieve among the highest encapsulation efficiencies in comparative analyses, although their industrial application remains limited relative to established drying technologies [29].

Overall, emerging encapsulation approaches reflect a shift from passive protection toward the rational engineering of delivery systems. Rather than maximizing oxidative stability alone, current developments increasingly seek to optimize both physicochemical stability and gastrointestinal functionality through precise control of particle architecture and interfacial organization.

### 3.5. Comparative Perspective on Technique Selection for Omega-3 Delivery Systems

Comparative evidence indicates that the performance of omega-3 encapsulation systems cannot be attributed to the encapsulation technique alone [5,30]. Instead, oxidative stability, encapsulation efficiency, surface oil content, and gastrointestinal performance emerge from the interplay between processing conditions, wall-material composition, emulsion characteristics, and the resulting microstructure. Consequently, encapsulation technologies should be viewed as alternative routes for engineering interfacial and matrix organization rather than as independent processing categories.

Across the available studies, a consistent trend is that systems with dense, continuous interfacial architectures generally exhibit lower surface oil levels and improved oxidative stability, regardless of the encapsulation method employed. For example, optimizing wall-material composition in spray-dried systems improved particle morphology and reduced lipid oxidation [33], whereas multilayer or double-coated structures, generated by combining complex coacervation with secondary coating, further reduced surface oil and enhanced barrier performance [32]. Likewise, reinforcement of protein–polysaccharide interfaces through natural polyphenols increased encapsulation efficiency and oxidative protection [55]. Collectively, these studies indicate that interfacial engineering is often more influential than the encapsulation technique itself. The same principle applies to gastrointestinal performance. Depending on their composition and organization, protein–polysaccharide interfaces may either delay lipid digestion by restricting enzyme diffusion or enhance bioaccessibility by maintaining fine droplet dispersion during digestion [5,27]. Similarly, improvements in precursor emulsion design, including optimized wall materials and intensified homogenization, have been shown to enhance both encapsulation efficiency and omega-3 bioaccessibility [15,56]. These observations indicate that delivery efficiency is governed not only by the initial capsule structure but also by its dynamic reorganization under gastrointestinal conditions.

Overall, no single encapsulation technique consistently outperforms all others. The most effective omega-3 delivery systems are those in which wall materials, interfacial architecture, particle morphology, and processing history are designed as an integrated system to balance oxidative protection with efficient lipid release and absorption [57]. This conclusion provides the rationale for the following sections, which focus on how protein–polysaccharide wall materials govern the structure–function relationships underlying encapsulation performance. Taken together, the available evidence suggests that future research should shift from comparing encapsulation techniques to understanding the structural principles that govern oxidative protection and gastrointestinal performance across different technologies. Future studies should report structural descriptors (e.g., surface oil, oxygen permeability, particle morphology) alongside conventional oxidation markers to facilitate meaningful comparison across encapsulation systems.

## 4. Wall Materials for Omega-3 Microencapsulation

The performance of omega-3 microencapsulation systems depends not only on the encapsulation technique but also on the physicochemical properties of the wall materials, which strongly influence particle formation and subsequent functionality [8,18,54]. Wall materials stabilize precursor emulsions, preserve interfacial integrity during particle formation, minimize surface oil and oxygen transport in the final structure, and, where required, regulate lipid release during gastrointestinal digestion [6,8,26,54]. Accordingly, their selection should be regarded as a multistage design process rather than a simple choice of carrier composition. From a mechanistic perspective, wall materials determine key structural attributes of encapsulated systems, including interfacial organization, matrix density, oxygen permeability, particle morphology, and gastrointestinal behaviour [50,54,56]. Their performance arises from interactions among biopolymers, lipids, and processing conditions, thereby making wall materials active structural components rather than passive coating agents [6,26]. Consequently, differences in oxidative stability, release behaviour, and lipid bioaccessibility are often governed as much by wall-material functionality as by the encapsulation technique itself [48,50]. Among the available materials, proteins and polysaccharides have received particular attention because they provide complementary functionalities within food-grade delivery systems. Proteins primarily contribute to emulsification and interfacial stabilization, whereas polysaccharides enhance viscosity, gel formation, matrix integrity, and barrier properties [8,50,54]. Their combination enables the formation of structured interfaces and matrices whose physicochemical properties can be tailored to improve both oxidative protection and gastrointestinal performance [6,50,56].

Consequently, understanding protein–polysaccharide interactions is essential for establishing structure–function relationships that support the rational design of advanced omega-3 delivery systems. Rather than acting as independent functional ingredients, proteins and polysaccharides should therefore be considered complementary structural elements whose interactions ultimately determine encapsulation performance. The following sections examine the major classes of wall materials used for omega-3 microencapsulation, with particular emphasis on how their interfacial and matrix-forming properties govern oxidative stability, digestion behaviour, and delivery efficiency.

### Selection Criteria for Wall-Materials

Emulsion-based microencapsulation of omega-3 oils requires wall materials to perform across two interconnected structural domains: the oil–water interface and the bulk matrix formed after particle solidification. The interfacial layer, established during emulsification, governs droplet stabilization through its composition, thickness, charge, and viscoelastic properties, whereas the bulk matrix provides the structural framework that immobilizes lipid droplets, limits oxygen transport, and preserves particle integrity during storage [58]. Because interfacial organization strongly influences subsequent matrix formation, these two domains should be considered as a continuous structural system rather than independent functional components.

Consequently, effective wall materials must simultaneously stabilize precursor emulsions, support cohesive particle formation during drying or gelation, minimize surface oil and oxygen permeability, and, when required, regulate lipid release during gastrointestinal digestion [8,21,58,59]. Beyond functional performance, successful food applications also require compatibility with processing conditions, sensory neutrality, scalability, regulatory acceptance, and the target food matrix [20].

From a formulation perspective, these requirements can be translated into three principal design objectives: (a) formation of physically stable emulsions with a controlled droplet-size distribution; (b) preservation of interfacial integrity during particle formation to minimize surface oil; and (c) development of dense matrices that restrict oxygen diffusion while maintaining adequate release characteristics. Their effectiveness is commonly assessed using parameters such as droplet-size stability, encapsulation efficiency, surface oil, and oxidative stability during storage [58,59]. Importantly, these parameters should not be interpreted in isolation, as interfacial modifications frequently propagate to particle morphology, storage stability, and gastrointestinal performance. Recent advances increasingly focus on engineering wall materials at the molecular level through protein–polysaccharide interactions, including electrostatic complexes and Maillard-type conjugates, which enable simultaneous tuning of interfacial architecture and matrix functionality beyond that achievable with simple physical blends [60]. These developments illustrate a broader shift from selecting individual wall materials toward engineering their molecular interactions to achieve predictable structure–function relationships. This variability is also evident in the studies summarized in Table 1, where differences in wall-material composition are associated with substantial differences in encapsulation efficiency, lipid loading, particle characteristics, and oxidative stability. Importantly, systems with comparable encapsulation efficiencies do not necessarily exhibit similar oxidative stability, further indicating that encapsulation efficiency alone is insufficient to describe the protective performance of a wall-material system.

## 5. Emulsion Stability and Interfacial Oxidation

The formulation stage preceding particle formation aims to produce a kinetically stable oil-in-water (O/W) emulsion that maintains a narrow droplet-size distribution and resists flocculation or coalescence before drying or gelation. Because each emulsion droplet serves as a template for the final particle, emulsion stability directly influences particle morphology, surface oil, and oxidative stability of the encapsulated system [58]. Consequently, the physicochemical properties of the precursor emulsion largely predetermine the structural organization and functionality of the final microcapsules.

Lipid oxidation in O/W emulsions is largely governed by interfacial phenomena, as the oil–water interface is the primary site where transition metals, reactive oxygen species, antioxidants, and lipid hydroperoxides interact [70,71]. Accordingly, interfacial composition, charge, thickness, and permeability regulate the transport of pro-oxidants and oxidation products, thereby influencing oxidation kinetics independently of fatty acid composition. The interface should therefore be regarded as a chemically active region rather than merely a physical boundary. Among the pro-oxidants involved, transition metals play a particularly important role. Iron-induced oxidation studies have shown that lipid stability depends not only on iron concentration but also on its localization and chemical speciation within the emulsion, emphasizing that oxidation is governed as much by the spatial organization of reactive species as by their abundance [72]. Likewise, immobilization of iron within alginate beads significantly reduced lipid oxidation compared with freely dissolved iron, demonstrating that spatial separation of catalytic metals from the oil–water interface can effectively suppress oxidation [73]. These observations suggest that oxidative stability is governed not only by the chemical composition of the emulsion but also by the spatial organization of pro-oxidants and antioxidants at the oil–water interface.

Collectively, these findings indicate that oxidation control relies not only on antioxidant activity but also on the regulation of the spatial distribution of pro-oxidants within the emulsion. Protein–polysaccharide interactions further determine interfacial stability through continuous restructuring of the adsorbed layer. Electrostatic deposition of polysaccharides such as fucoidan or gum arabic onto protein-coated droplets modifies interfacial thickness, charge, and barrier properties, thereby improving both oxidative and emulsion stability [70,71,74]. The effectiveness of these structures additionally depends on protein characteristics, including solubility, conformational flexibility, aggregation state, and proximity to the isoelectric point, all of which govern adsorption kinetics and film formation [75]. Consequently, effective interfacial stabilization arises from controlled protein adsorption and rearrangement, cohesive interfacial film formation, and protein–polysaccharide interactions, which collectively enhance droplet stability [76]. This illustrates that interfacial engineering begins at the emulsion stage and strongly influences the structural quality of the final encapsulated system.

### 5.1. Drying, Matrix Formation, and Oxygen Transport

During spray drying, rapid water removal generates concentration gradients within the droplets, promoting early skin formation followed by consolidation into a continuous solid matrix. As dehydration proceeds, increasing solids concentration reduces molecular mobility and immobilizes dispersed lipid droplets within the developing structure [8]. The effectiveness of encapsulation, therefore, depends on the ability of wall materials to simultaneously preserve interfacial integrity during drying and generate a dense matrix that minimizes oil migration toward the particle surface. Because surface oil is directly exposed to atmospheric oxygen, its reduction is generally associated with improved oxidative protection in omega-3 powders [58].

Drying conditions can substantially modify the resulting matrix architecture and, consequently, the extent of oxygen exposure of encapsulated lipids. Anwar and Kunz [77] demonstrated pronounced structural differences among fish-oil microcapsules produced by spray granulation (SG), SD and FD. SG generated large, multilayer particles, whereas SD produced smaller, partially collapsed particles and FD resulted in irregular, highly porous structures with pores extending into the particle interior. These differences were accompanied by surface oil contents of 0.363%, 2.627%, and 11.833% for SG, SD, and FD, respectively. During eight weeks of storage at room temperature, peroxide values remained below 20 meq/kg oil for most SG formulations, whereas maximum values of 151.65 ± 10.03 and 390.38 ± 31.44 meq/kg oil were observed for SD and FD systems, respectively. The particularly poor stability of the freeze-dried systems was attributed to their porous architecture, which facilitated oxygen penetration into the matrix. The influence of drying-induced architecture was further demonstrated by Ramos et al. [78] in fish-oil powders produced by conventional spray drying and vacuum spray drying (VSD). Conventional spray drying at an inlet temperature of 190 °C generated particles with a relatively large central vacuole and a thinner matrix wall, whereas VSD at approximately 30 °C produced particles with smaller central vacuoles and thicker walls. Surface oil decreased from 4.45 ± 0.43% to 1.95 ± 0.25%, while encapsulation efficiency increased from 69.22 ± 5.66% to 87.21 ± 0.67% with VSD. These structural differences were accompanied by an approximately 11-fold increase in Rancimat induction time, from 0.06 h for conventionally spray-dried particles to 0.67 h for VSD particles.

Beyond morphological observations, direct measurements of oxygen transport provide further evidence that capsule architecture influences oxygen accessibility. Using electron spin resonance, Rahmani-Manglano et al. [59] showed markedly different oxygen equilibration kinetics among capsules produced by different encapsulation routes. Spray-dried particles, approximately 90% of which were smaller than 20 µm, required approximately 2 h to reach equilibrium following oxygen exposure, whereas monoaxially electrosprayed particles, approximately 90% of which were smaller than 1.5 µm, reached equilibrium within approximately 10 min. The spray-dried system also exhibited a four-day oxidative lag phase during storage at 25 °C. The authors associated the slower oxygen transport with differences in particle dimensions and diffusion pathways, illustrating that oxygen accessibility can differ markedly among capsule architectures and can coincide with different oxidative behavior.

Importantly, however, structural descriptors such as encapsulation efficiency and surface oil should not be interpreted as isolated predictors of oxidative stability. Rahmani-Manglano et al. [79] obtained encapsulation efficiencies above 91% and similar particle sizes of approximately 12–14 µm across spray-dried fish-oil formulations, yet their oxidative behavior differed substantially depending on antioxidant location within the particle. After six weeks of storage at 25 °C, the nonanal signal was approximately 46.000 for microcapsules containing a hydrophilic rosemary extract, compared with approximately 3.500 for particles containing amphiphilic whey-protein hydrolysate. The authors attributed this difference to antioxidant partitioning: the hydrophilic antioxidant was trapped within the glassy carbohydrate matrix, whereas the amphiphilic hydrolysate was located closer to the oil-matrix interface, where oxidation-related reactions occur. Thus, oxidative protection depends not only on bulk retention of oil but also on the spatial organization of lipids and protective components within the matrix.

Importantly, oxidation may also modify the encapsulation structure itself. Lipid-protein co-oxidation and other interfacial reactions can alter film composition and integrity, thereby influencing droplet interactions and oil redistribution during processing and storage [3,71]. Oxidative stability should therefore be viewed as a dynamic property emerging from continuous interactions between chemical reactions and structural evolution rather than as a fixed consequence of the initial particle architecture.

### 5.2. Powder State Effects on Stability

In emulsion-based omega-3 encapsulation, powder properties strongly influence oxidative and physical stability during storage. Key parameters include moisture content, water activity, hygroscopicity, caking tendency, flowability, and dispersibility, which collectively affect powder quality and storage performance [54]. Moisture content and water activity are particularly important because they influence both the physical stability and oxidative behaviour of encapsulated lipid powders [50,54].

A critical determinant of stability is the physical state of the amorphous matrix. Water acts as a plasticizer, reducing the glass-transition temperature and increasing molecular mobility, thereby promoting stickiness, caking, oxygen diffusion, and lipid redistribution. As a result, oxidation may accelerate as the matrix shifts from a glassy to a rubbery state. Glass-transition-based models have therefore been proposed to predict powder stability and optimize storage conditions for spray-dried food systems [80]. Collectively, these observations highlight that powder stability is directly linked to chemical shelf-life rather than merely to handling properties. Powder characteristics also influence reconstitution behaviour. Efficient wetting and redispersion require appropriate surface composition and controlled porosity; however, excessive porosity may simultaneously increase oxygen accessibility during storage. Consequently, wall-material design must balance storage stability with reconstitution performance, for example, by minimizing surface oil while maintaining adequate dispersibility [58,59]. Overall, the long-term stability of omega-3 powders stems from the combined effects of matrix physical state, environmental humidity, and oxygen transport, illustrating that wall-material selection ultimately governs storage performance by influencing matrix architecture.

These observations reinforce the concept that the functionality of wall materials is determined not only by their individual properties but also by the molecular interactions established between proteins and polysaccharides. The following section, therefore, examines these interactions as the mechanistic basis for the design of advanced omega-3 delivery systems.

### 5.3. Protein–Polysaccharide Interactions and Oxidative Stability in Omega-3 Microencapsulation Systems

Protein–polysaccharide interactions are central to the design of omega-3 delivery systems because they determine interfacial organization, matrix formation, and, ultimately, the oxidative stability and digestive behaviour of encapsulated lipids [39,56]. Their effects extend throughout the encapsulation process, from stabilization of the parent oil-in-water emulsion to formation of the final dried or gelled structure. Consequently, molecular interactions established at the emulsion stage largely predetermine particle morphology, oxygen transport, and lipid accessibility in the final delivery system [3,4,70].

Most food-grade protein–polysaccharide systems are governed by non-covalent interactions, primarily electrostatic attraction, hydrogen bonding, and hydrophobic interactions [34,66]. The relative contribution of these forces depends on formulation variables such as pH, ionic strength, biopolymer ratio, and molecular conformation. Depending on these conditions, proteins and polysaccharides may assemble into soluble complexes, multilayer interfacial structures, or phase-separated coacervates, each providing distinct structural and barrier properties [6,26,81].

From a mechanistic perspective, oxidative stability should therefore be regarded as an emergent property of the entire encapsulation system rather than of individual wall materials [70]. Molecular interactions govern interfacial architecture, matrix organization, and oxygen transport, thereby determining lipid protection during storage and behaviour under gastrointestinal conditions. The following sections examine the major interaction mechanisms and discuss how they contribute to the structure–function relationships governing omega-3 microencapsulation.

### 5.4. Protein–Polysaccharide Interactions as Structural Determinants of Omega-3 Encapsulation

#### 5.4.1. Electrostatic and Polyelectrolyte Complexation

Electrostatic interactions are the principal driving force for protein–polysaccharide association in most food-grade encapsulation systems. A major advantage of electrostatic interactions is that complex formation can be precisely regulated through pH and ionic strength. Proteins exhibit pH-dependent surface charge, whereas many polysaccharides used as wall materials contain negatively charged functional groups, promoting adsorption onto protein-coated oil droplets and the formation of structured interfacial layers [70,74].

The behaviour of these systems depends not only on the net charge of the biopolymers but also on the distribution of charged residues on the protein surface. Even near the isoelectric point, localized positively charged domains may remain available for polysaccharide binding, thereby allowing the formation of thicker interfacial layers with modified ζ-potential, steric stabilization, and barrier properties [70]. Electrostatic interactions contribute to structural organization at both the interfacial and bulk levels. At the oil–water interface, they promote multilayer film formation, whereas in the continuous phase, they may generate soluble complexes or, under near-charge-neutral conditions, induce liquid–liquid phase separation, leading to complex coacervation [6,81]. Interfacial complexation generally improves emulsion stability and limits oil migration during drying, thereby reducing surface oil, a major determinant of oxidation in spray-dried omega-3 powders [58,69]. By contrast, excessive charge neutralization may reduce electrostatic repulsion and increase susceptibility to flocculation unless rapid shell formation stabilizes the droplets. Ionic strength further modulates this balance by screening electrostatic interactions, thereby altering complexation behaviour and the resulting barrier architecture.

Electrostatic organization also influences oxidative stability and lipid digestion. Because the oil–water interface is the primary site where aqueous pro-oxidants interact with lipid substrates, the interfacial layer’s charge and permeability influence the transport and localization of catalytic metal ions, including iron [70,72]. At the same time, thicker protein–polysaccharide interfacial layers may delay displacement by bile salts during digestion, thereby modifying lipolysis and lipid bioaccessibility [3,4]. These observations highlight that electrostatic interactions simultaneously govern colloidal stability, oxidative protection, and gastrointestinal performance by controlling the structural organization of the oil–water interface. Despite these advances, the quantitative relationships between interfacial architecture and oxidative stability remain insufficiently understood, limiting the rational design of protein–polysaccharide delivery systems.

#### 5.4.2. Hydrogen Bonding and Hydrophobic Contributions

When electrostatic interactions are weakened, for example, by charge screening or near the protein isoelectric point, hydrogen bonding and hydrophobic interactions become increasingly important in stabilizing protein–polysaccharide assemblies. Hydrogen bonds create multipoint contacts between biopolymers, reinforcing interfacial layers and promoting the formation of cohesive matrix networks during particle formation. Their structural importance is illustrated by WPI-flaxseed gum coacervates, where disruption of hydrogen bonding significantly altered rheological behaviour [43].

Hydrogen bonding becomes particularly relevant during dehydration, when increasing biopolymer concentration promotes closer molecular contacts and matrix consolidation. The resulting reduction in molecular mobility and lipid migration may improve oxidative stability by limiting oxygen diffusion and reducing oil redistribution within the dried particle [69]. Hydrophobic interactions provide a complementary stabilization mechanism at the oil–water interface. Owing to their amphiphilic nature, proteins adsorb rapidly at the interface, whereas their surface hydrophobicity influences adsorption kinetics, interfacial rearrangement, and film cohesion. Processing conditions strongly affect these properties; for example, moderate ultrasound treatment of soy protein isolate increased surface hydrophobicity and improved emulsifying performance, whereas excessive aggregation impaired interfacial stabilization [82]. Certain polysaccharides also exhibit amphiphilic characteristics and may further enhance interfacial stability under processing stress [83]. Together, electrostatic interactions, hydrogen bonding, and hydrophobic interactions cooperate to generate robust interfacial structures that resist coalescence and dehydration. These mechanisms are also relevant in Pickering emulsions, where particle wettability governs adsorption at the interface. Protein–polysaccharide complexation can optimize particle wettability and thereby improve emulsion stability [84]. However, increased hydrophobicity alone does not guarantee stronger interfacial films, because interfacial elasticity also depends on the ability of adsorbed particles to form cohesive networks [85].

The functional importance of hydrogen bonding and hydrophobic interactions is evident in their contribution to the oxidative stability of omega-3 delivery systems. DHA-rich algal oil Pickering emulsions stabilized by WPI-pectin complexes showed enhanced resistance to oxidation [86], while modification of whey-protein interfacial organization with sesamol improved the oxidative stability of fish-oil emulsions [14,87]. These findings highlight that hydrogen bonding and hydrophobic interactions complement electrostatic forces in governing interfacial cohesion, barrier properties, and oxidative protection.

#### 5.4.3. Influence of Environmental and Processing Conditions

While molecular interactions determine the potential for protein–polysaccharide association, environmental conditions govern the structures ultimately formed. Among these, pH is the principal control parameter because it regulates protein charge, polysaccharide ionization, and the balance between interfacial complexation and bulk phase separation. Consequently, pH largely determines whether the system forms soluble complexes, multilayer interfacial assemblies, or phase-separated coacervates, each exhibiting distinct structural and functional properties [6,81].

In many protein-polyanion systems, decreasing pH strengthens electrostatic attraction until charge compensation is reached, promoting complex coacervation. However, reduced electrostatic repulsion may simultaneously destabilize emulsions if shell formation does not occur rapidly enough [81]. This behaviour is particularly evident in plant-protein systems, where the pH range allowing both sufficient solubility and effective complexation is often narrow. For example, rice protein-gum arabic emulsions exhibited pronounced pH-dependent changes in structure and stability [88]. In addition to pH, ionic strength provides another important means of controlling protein–polysaccharide interactions. Electrostatic screening modifies both attractive and repulsive forces, allowing the same formulation to exist as a stable dispersion, a flocculated system, or a phase-separated coacervate depending on salt concentration [70,81]. Because destabilization before drying promotes droplet growth and surface oil formation, environmental conditions established during formulation directly influence the oxidative stability of the final powder [6,60].

Processing conditions further influence the architecture of protein–polysaccharide assemblies. Heat treatment, shear, and mixing sequence may alter protein conformation, changing the accessibility of charged and hydrophobic domains and thereby modifying interfacial organization [89]. As a result, identical formulations may generate different microstructures depending on their processing history, highlighting that encapsulation performance depends not only on formulation composition but also on the pathway through which the final structure is assembled. These observations illustrate that environmental and processing conditions should be regarded as structural design variables rather than merely operational parameters, because they ultimately determine the architecture and functionality of protein–polysaccharide delivery systems.

#### 5.4.4. Destabilization Pathways: Depletion and Bridging Flocculation

Despite careful formulation, protein–polysaccharide emulsions may undergo destabilization when attractive interactions overcome colloidal repulsion. Such processes are particularly important before drying because droplet aggregation promotes oil migration toward particle surfaces, increases surface oil, and ultimately reduces oxidative stability of the resulting powder [59,60].

Two polymer-mediated mechanisms are especially relevant. Depletion flocculation occurs when non-adsorbing polymers remain in the continuous phase, generating an osmotic attraction between droplets as described by the Asakura-Oosawa theory [90]. The extent of this attraction depends on polymer concentration and conformation, indicating that increased viscosity does not necessarily improve emulsion stability [91]. By contrast, bridging flocculation arises when partially adsorbed polymer chains simultaneously bind multiple droplets, generating interconnected aggregates. Zhai et al. [92] demonstrated that depletion and bridging flocculation produced distinct aggregate structures and differently affected lipid digestion in WPI-stabilized emulsions. These mechanisms therefore influence not only emulsion stability but also interfacial accessibility and the subsequent functionality of encapsulated omega-3 systems.

Although generally undesirable, controlled destabilization may also be exploited to engineer structured colloidal networks. For example, depletion interactions have been used to generate Pickering high-internal-phase emulsions with improved structural integrity while maintaining resistance to coalescence through particle armoring [93]. Thus, controlled colloidal interactions can, under appropriate conditions, be harnessed to create functional delivery structures rather than simply causing emulsion failure.

#### 5.4.5. Functional Consequences for Omega-3 Delivery

The structural organization generated by protein–polysaccharide interactions ultimately determines the functional performance of omega-3 delivery systems by controlling both oxidative stability during storage and lipid accessibility during digestion. The stability of the precursor emulsion influences droplet size distribution and aggregation, thereby affecting oil migration, surface oil formation, oxygen transport, and matrix continuity in the final encapsulated system [58].

Oxidative stability is further governed by interfacial chemistry. Oxidation in Fe^2+^-containing emulsions has been linked to the transport of metal complexes toward the oil–water interface, demonstrating that oxidation depends not only on lipid composition but also on interfacial organization and aqueous-phase chemistry [70,72]. During digestion, the same interfacial structures regulate enzyme accessibility and lipolysis. Interfacial composition, droplet aggregation, and susceptibility to bile-salt displacement determine the extent of lipid digestion, with bridging-type aggregates generally restricting lipase access more effectively than depletion-type aggregates [92]. Consequently, oxidative stability and lipid bioaccessibility should not be regarded as independent formulation objectives, since both emerge from the same interfacial architecture [3,70].

Overall, protein–polysaccharide interactions constitute the mechanistic link between molecular design and delivery performance. By governing interfacial architecture, matrix organization, and transport processes, they simultaneously determine oxidative protection, gastrointestinal functionality, and ultimately the overall performance of omega-3 delivery systems.

### 5.5. Structural Determinants of Oxidative Stability During Microencapsulation

The oxidative stability of omega-3-rich oils is not an intrinsic property of the lipid itself but an emergent consequence of the microstructure generated during encapsulation. As the system evolves from an oil-in-water emulsion to a dried or gelled particle, the dominant oxidation environment changes together with the spatial organization of lipids, pro-oxidants, and oxygen transport pathways. In emulsions, oxidation is governed primarily by events occurring at the oil–water interface, whereas in dried particles it is governed mainly by lipid distribution, surface oil content, matrix continuity, and oxygen permeability [94,95].

This structural evolution also changes the mechanisms governing oxidation. During emulsification, interfacial composition determines the accessibility of transition metals, reactive oxygen species, and antioxidants to the lipid phase. Following drying or gelation, the continuity and permeability of the encapsulating matrix, together with molecular mobility within the particle, become the dominant determinants of oxidation kinetics. Consequently, identical omega-3 oils may exhibit markedly different oxidative behaviour depending on the structural architecture of the delivery system into which they are incorporated [70,96].

From this perspective, oxidative stability should be regarded as an emergent property of the entire delivery system, arising from the combined effects of interfacial organization, matrix architecture, and transport phenomena that collectively define the oxidative environment experienced by encapsulated lipids.

#### 5.5.1. Critical Sites of Oxidation Initiation

In omega-3 delivery systems, the dominant site of lipid oxidation changes as the encapsulation process progresses. In oil-in-water emulsions, oxidation is initiated primarily at the oil–water interface, where unsaturated lipids, dissolved oxygen, transition metals, and other pro-oxidants coexist within a confined reaction zone [97]. Consequently, interfacial composition, charge, thickness, and permeability largely determine the transport of reactive species to the lipid phase.

Transition metal ions, particularly iron, are among the principal catalysts of oxidation in emulsified systems. Their pro-oxidant activity depends not only on concentration but also on chemical speciation and interfacial localization. Experimental studies have shown that interfacial charge and metal-ligand interactions influence the distribution of iron complexes near the droplet surface, thereby modifying oxidation kinetics [72]. Proteins adsorbed at the interface further contribute to oxidation control by forming diffusion barriers and, in some cases, acting as preferential targets for radical reactions, thereby partially protecting the encapsulated lipids [98].

Following drying, the dominant oxidation sites shift from the interface to the particle microstructure. Surface oil becomes the most oxidation-prone fraction because of its direct exposure to atmospheric oxygen, whereas oxidation of encapsulated lipids is largely governed by oxygen diffusion through the surrounding matrix [94,99]. Consequently, oxidation during storage depends less on encapsulation efficiency itself than on matrix continuity, oxygen permeability, and lipid distribution within the particle.

These observations highlight that oxidation initiation is governed by the evolving microstructure of the delivery system. As the system transforms from an emulsion into a solid particle, the dominant oxidation environment shifts from interfacial reactions to transport-controlled processes within the encapsulating matrix, illustrating how structural evolution governs oxidative stability throughout encapsulation and storage.

#### 5.5.2. Surface Oil, Matrix Discontinuities, and Oxygen Transport

Among the structural determinants of oxidative stability, surface oil is considered one of the strongest predictors of oxidation in encapsulated omega-3 systems. Because this lipid fraction remains directly exposed to atmospheric oxygen, it oxidizes substantially faster than oil embedded within the particle matrix and therefore frequently represents the primary site of oxidation initiation during storage [94].

However, oxidative stability cannot be explained by surface oil alone. Once oxygen penetrates the encapsulating matrix, oxidation may also occur within the particle interior. Consequently, the effectiveness of encapsulation depends largely on the matrix’s barrier properties, including its continuity, density, and oxygen permeability [99]. Structural defects such as pores, fissures, and microcracks shorten diffusion pathways, facilitating oxygen transport toward encapsulated lipid domains and accelerating oxidation. Particle geometry further modulates these transport processes. Smaller particles generally possess a higher surface-to-volume ratio and shorter oxygen diffusion pathways, making them more susceptible to oxidative deterioration [100]. Likewise, heterogeneity in particle size and morphology may create local differences in oxygen accessibility and oxidation behaviour within the same powder.

These observations indicate that oxidation in dried omega-3 delivery systems is fundamentally a transport-controlled phenomenon. Matrix architecture determines how oxygen reaches the lipid phase, whereas the spatial distribution of surface and encapsulated oil determines where oxidation is initiated and how it subsequently propagates throughout the particle. Consequently, improving oxidative stability requires engineering oxygen transport pathways rather than simply minimizing surface oil.

#### 5.5.3. Metal-Catalysed Oxidation and Aqueous-Phase Chemistry

Transition metals, particularly iron and copper, are among the most effective catalysts of lipid oxidation in omega-3 emulsions. Their pro-oxidant activity depends not only on concentration but also on chemical speciation and localization within the system [72]. In emulsified systems, metal ions frequently form complexes with ligands present in the aqueous phase, and the properties of these complexes largely determine their transport toward the oil–water interface, where oxidation is initiated.

Consequently, aqueous-phase chemistry plays a central role in oxidation control. Buffer composition, chelating agents, and other ligands can either reduce or enhance oxidation by modifying the distribution of catalytically active metal species between the continuous phase and the interface. For example, phytic acid generally inhibits oxidation through metal chelation, although under certain conditions it may promote the accumulation of metal complexes near positively charged interfacial layers [101]. Likewise, protein hydrolysates may improve oxidative stability by combining emulsifying, metal-chelating, and radical-scavenging activities [102].

The influence of aqueous-phase chemistry extends beyond the emulsion stage. Oxidation initiated before drying generates lipid hydroperoxides that may continue to decompose during storage, even after encapsulation. Consequently, oxidative stability depends not only on the barrier properties of the dried matrix but also on the chemical history of the precursor emulsion. Effective control of metal contamination and aqueous-phase composition should therefore be regarded as an integral component of omega-3 encapsulation design, as they define the oxidative environment from which the final delivery system is formed.

### 5.6. Storage Conditions as Determinants of Stability

The oxidative stability of microencapsulated omega-3 oils is determined not only by the initial capsule structure but also by the environmental conditions encountered during storage. Temperature, oxygen availability, light exposure, and water activity collectively influence oxidation kinetics and modify the physical state of the encapsulating matrix. Consequently, storage stability reflects the combined effects of chemical reactions and continuous structural evolution rather than oxidation alone. Oxidative deterioration is spatially heterogeneous within encapsulated particles. Surface lipid remains the most reactive fraction because of its direct exposure to oxygen, whereas oxidation of encapsulated oil depends on oxygen diffusion through the surrounding matrix. Matrix continuity, porosity, and lipid distribution therefore govern oxygen transport to the particle interior and, consequently, the propagation of oxidation during storage [103,104,105].

Storage also induces progressive physical aging of the encapsulating matrix. Moisture sorption, matrix plasticization, caking, and oil migration may reduce barrier integrity, increase oxygen permeability, and accelerate oxidation over time [106,107,108]. Thus, long-term stability should be regarded as a dynamic consequence of coupled physical and chemical processes, whereby continuous changes in matrix structure progressively modify the oxidative behaviour of encapsulated omega-3 lipids. These observations indicate that storage conditions should not be regarded merely as external factors affecting oxidation, but as variables that continuously reshape the structural architecture governing oxygen transport and lipid stability.

#### 5.6.1. Temperature and Light

Temperature is a major determinant of the oxidative stability of encapsulated omega-3 oils because it accelerates both lipid oxidation and oxygen transport within the encapsulating matrix. Besides accelerating oxidation kinetics, elevated temperatures enhance molecular mobility and may induce matrix relaxation, progressively weakening barrier properties and facilitating oxygen diffusion toward encapsulated lipid domains [109,110]. The extent of thermal protection depends strongly on capsule architecture. Dense, continuous matrices generally offer greater resistance to oxygen transport than porous structures, resulting in slower oxidation during storage. Accordingly, spray-dried powders often exhibit higher oxidative stability than freeze-dried systems because their denser microstructure provides a more effective barrier to oxygen diffusion [111,112].

Light exposure represents an additional oxidation pathway, particularly via singlet-oxygen-initiated photo-oxidation. Unlike radical autoxidation, this mechanism proceeds independently of thermal activation and can rapidly generate volatile oxidation products that contribute to sensory deterioration. Protein-containing wall systems often provide greater resistance to photo-oxidation than carbohydrate-based matrices, reflecting differences in interfacial composition and light-shielding properties [113]. Consequently, protection against both temperature- and light-induced oxidation should be considered an integral component of the design and storage strategy for omega-3 delivery systems.

#### 5.6.2. Water Activity and Moisture Sorption

Water activity is a key determinant of the physical stability of encapsulated omega-3 powders because it governs molecular mobility within the matrix. Unlike moisture content, water activity reflects the availability of water for physicochemical processes and therefore influences matrix plasticization, glass transition, and oxygen transport [23]. As water activity increases, the encapsulating matrix becomes progressively plasticized, reducing its barrier performance and facilitating oxygen diffusion. In rice protein-based flaxseed oil microcapsules, higher water activity promoted caking, particle agglomeration, and structural rearrangements that were associated with reduced storage stability [106]. These changes may also increase lipid mobility and promote migration of encapsulated oil toward the particle surface. The relationship between water activity and oxidation is not strictly linear. Maximum oxidation rates are frequently observed at intermediate water activity, where molecular mobility is sufficient to sustain radical reactions while oxygen remains readily available for diffusion [114]. By contrast, oxidation may be limited at very low water activity due to restricted molecular mobility and at high water activity due to changes in oxygen transport and partitioning.

Moisture-induced structural relaxation may further increase surface oil by promoting matrix defects and lipid migration, thereby accelerating oxidation during storage [107,108]. Overall, these findings indicate that water activity regulates oxidative stability primarily through its effects on the matrix’s physical state and oxygen transport, rather than through direct chemical interactions with the lipid phase.

#### 5.6.3. Oxygen Availability and Packaging

Oxygen availability is a primary determinant of oxidative stability in encapsulated omega-3 systems, as it directly governs lipid autoxidation. Although a small amount of oxygen may remain entrapped within the powder after processing, studies indicate that oxygen diffusing from the surrounding atmosphere is the dominant driver of oxidation during storage [115]. Consequently, long-term oxidative stability depends largely on controlling oxygen ingress rather than solely minimizing residual oxygen within the particles. The importance of environmental oxygen exposure is supported by quantitative observations in spray-dried fish-oil systems. Linke et al. [115] demonstrated that non-encapsulated surface oil oxidized on average 7.4 ± 2.0 times faster than the encapsulated lipid fraction when the proportion of free oil was below 7.86%. Nevertheless, hydroperoxide concentrations in the encapsulated oil reached approximately 30–130 mmol/kg oil, corresponding to 6–26 mmol bound O_2_/kg oil and substantially exceeding the 0.76–1.25 mmol O_2_/kg oil initially dissolved in marine oil. These findings demonstrate that the encapsulation matrix slows direct lipid exposure but does not completely prevent environmental oxygen from reaching the internal lipid fraction.

Packaging, therefore, represents an integral component of oxidation control. Vacuum packaging and storage under inert atmospheres effectively reduce oxygen exposure and delay the accumulation of primary and secondary oxidation products compared with conventional atmospheric storage [116]. Likewise, packaging permeability and headspace oxygen concentration strongly influence oxidation during shelf life. This effect was quantitatively demonstrated by Novaes et al. [117], who compared commercial packaging systems for milk powder enriched with microencapsulated fish oil. Oxygen transmission rates ranged from <0.005 cm^3^/package day for metal and composite cans to 0.731 ± 0.06 cm^3^/m^2^·day for PETmet/LDPE and 1197 ± 10.53 cm^3^/m^2^·day for BOPP. After 15 weeks at 43 °C, headspace oxygen decreased from approximately 20.7% to 0.40 ± 0.27% in metal cans and 2.17 ± 0.15% in composite cans, whereas it remained at 20.42 ± 0.32% in BOPP/PP because oxygen ingress through the highly permeable material continuously replenished the headspace [117]. The practical relevance of these differences was confirmed by Dantas et al. [118] during long-term storage, who evaluated the same types of packaging for 360 days at 25 °C and 75% relative humidity. At the end of storage, EPA + DHA contents were 392.6 and 390.3 mg/100 g in metal and composite cans, respectively, compared with 374.2 mg/100 g in PETmet/LDPE and 352.0 mg/100 g in the highly oxygen-permeable BOPP/PP system [118]. Thus, packaging permeability determines whether oxygen consumed in oxidative reactions is continuously replenished from the surrounding atmosphere, which can influence the long-term retention of encapsulated omega-3 fatty acids.

Beyond passive protection, active packaging technologies incorporating oxygen scavengers have emerged as an additional strategy for prolonging storage stability. Iron-based scavengers can effectively reduce residual oxygen within sealed systems, while multilayer packaging materials with low oxygen permeability provide additional protection against external oxygen ingress [119,120]. More recently, Kurek et al. [121] evaluated chitosan–gelatin films containing gallic acid and orange essential oil to protect fish oil during 30 days of storage. The combined active film exhibited 88.08 ± 0.25% DPPH radical-inhibition activity, while the K_232_ value of the protected oil after 30 days was 8.54, compared with 12.94 in the uncovered control. Film-protected samples exhibited significantly lower K_232_ values than the uncovered control from day 7 to day 30 (*p* < 0.05) [121]. These findings indicate that packaging can contribute to oxidative protection through both passive limitation of oxygen transfer and active antioxidant functionality.

### 5.7. Assessment and Comparison of Oxidative Stability Across Microencapsulation Systems

Direct comparison of oxidative stability among omega-3 microencapsulation systems remains challenging because oxidation is governed by the combined effects of formulation, microstructure, processing history, and storage conditions [48,59,70]. Consequently, differences in oxidation markers reported across studies cannot be interpreted independently of the structural characteristics of the encapsulation system. Identical peroxide values or secondary oxidation products may therefore reflect different oxidation kinetics or rate-limiting steps depending on particle architecture, oxygen transport, and lipid distribution [59,73].

Meaningful evaluation of encapsulation performance, therefore, requires integrating conventional oxidation markers with structural descriptors of the delivery system [9,54]. Alongside peroxide value and secondary oxidation products, structural descriptors such as surface oil content, oxygen permeability, particle morphology, and matrix physical state should also be considered when assessing oxidative stability [6,48,50,59]. This integrated approach enables more reliable interpretation of the relationships between formulation, structure, and oxidation behaviour across different encapsulation technologies. Overall, oxidative stability should be regarded not as a single analytical endpoint but as an emergent structure–function property arising from the interplay between lipid chemistry, matrix architecture, and oxygen transport throughout processing and storage [59,70].

Reliable assessment of oxidative stability requires combining analytical markers that represent different stages of lipid oxidation. Peroxide value (PV) is the most widely used indicator of primary oxidation because it reflects hydroperoxide formation during the early stages of autoxidation [25,77]. However, because hydroperoxides are unstable intermediates, PV alone cannot adequately describe the progress of oxidation. For this reason, PV is commonly complemented by markers of secondary oxidation, including *p*-anisidine value (*p*-AV) and thiobarbituric acid reactive substances (TBARS), with the latter primarily reflecting the formation of malondialdehyde and related secondary lipid oxidation products [4,122]. Analysis of volatile compounds, such as propanal and hexanal, provides additional information on advanced oxidation and shelf-life development in encapsulated systems [77]. Retention of nutritionally relevant fatty acids, particularly EPA and DHA, provides a direct measure of functional protection, whereas kinetic parameters such as induction time or oxidative stability index (OSI) offer complementary information on oxidation resistance under standardized conditions [42,123,124,125]. From a practical perspective, lower PV, *p*-AV, TBARS, and volatile oxidation-product levels measured at the same storage time and under identical conditions indicate better oxidative protection, whereas longer induction times or higher OSI values indicate greater resistance to oxidation. Reference limits are available for bulk oils. For fish oils, Codex CXS 329-2017 specifies maximum values of 5 meq O_2_/kg oil for PV, 20 for *p*-anisidine value, and 26 for total oxidation value (TOTOX = 2PV + *p*-AV) [126]. For refined vegetable oils, Codex CXS 210-1999 specifies a PV of up to 10 meq O_2_/kg oil, while values up to 15 meq O_2_/kg oil are indicated for cold-pressed and virgin oils [127]. However, these limits should be regarded only as contextual reference values for microencapsulated oils, particularly when accelerated oxidation protocols are used. In such systems, within-study comparisons performed under identical storage and analytical conditions are generally more meaningful than direct comparison with a universal threshold. Accordingly, no single oxidation marker should be interpreted in isolation, and assessment should combine primary and secondary oxidation indices with kinetic and structural information.

Chemical indicators become substantially more informative when interpreted alongside structural characteristics of the encapsulation system. Among these descriptors, surface oil is one of the strongest predictors of oxidative stability because lipids exposed at the particle surface remain readily accessible to oxygen [77,122]. The matrix architecture further determines oxidation behavior by controlling oxygen transport. Parameters such as particle size, porosity, glass transition temperature, and matrix continuity influence oxygen permeability and diffusion pathways, thereby governing oxygen access to encapsulated lipid domains [103]. These structural characteristics evolve during storage as moisture sorption, matrix plasticization, and oil migration progressively modify barrier properties.

Consequently, reliable comparison of microencapsulation systems requires integrating chemical oxidation markers with structural descriptors. Oxidative stability should therefore be regarded as a structure-dependent property arising from the interplay between interfacial organization, matrix architecture, and oxygen transport. Accordingly, structural descriptors should be considered indispensable complements to conventional oxidation markers for reliable comparison of omega-3 microencapsulation systems.

## 6. Gastrointestinal Functionality of Microencapsulated Omega-3 Delivery Systems

The gastrointestinal functionality of microencapsulated omega-3 systems is determined by a sequence of structural transformations that occur during digestion, rather than by the initial properties of the encapsulated powder or emulsion alone. Processing and wall-material selection establish the initial droplet size, interfacial composition, matrix density, and resistance to aggregation, whereas subsequent exposure to gastric acid, pepsin, intestinal pH, bile salts, and pancreatic enzymes progressively reorganizes these structures [128,129]. These transformations regulate the accessibility of the lipid phase to digestive enzymes and therefore determine lipid release, lipolysis, and ultimately the fraction of omega-3 fatty acids incorporated into mixed micelles and potentially available for intestinal absorption [130,131]. Accordingly, gastrointestinal performance should be interpreted through a processing–structure–function framework in which the protective structure generated during encapsulation undergoes stage-specific modification during gastrointestinal transit.

### 6.1. Structural Transformations During Gastrointestinal Digestion

The gastric phase represents the first major structural challenge for encapsulated omega-3 systems. Acidification alters the ionization state and electrostatic interactions of proteins and polysaccharides, while pepsin-mediated proteolysis can partially or completely disrupt protein-containing interfacial layers. Depending on wall composition, these changes may induce swelling, erosion, flocculation, or coalescence, thereby modifying the lipid-water interfacial area subsequently available for intestinal digestion. Protein–polysaccharide interactions can counteract these effects by increasing steric and electrostatic stabilization or by forming matrices that remain comparatively resistant to gastric degradation. This effect is particularly evident in complex coacervate systems. Timilsena et al. [132] demonstrated that chia-oil microcapsules formed from chia protein isolate-chia seed gum (CPI-CSG) complex coacervates exhibited substantially greater gastric resistance than systems containing the individual biopolymers. The CPI-CSG formulation achieved an encapsulation efficiency of 93.9% and released only 37.9% of the encapsulated oil during gastric digestion, compared with 79.1% for CPI-based microcapsules and 84.3% for the corresponding emulsified oil. However, this increased structural resistance persisted into the intestinal phase, reducing FFA release to 583.4 mg/g oil, compared with 967.3 mg/g for unencapsulated oil. Thus, the dense, electrostatically assembled coacervate structure protected the lipid core against premature gastric release while simultaneously reducing subsequent access by pancreatic lipase.

A more pronounced stage-specific response was observed in spray-dried DHA-rich algae-oil microcapsules formulated with different wall materials) [109]. Despite similarly high encapsulation efficiencies (>98%), wall composition strongly affected gastrointestinal behavior. Microcapsules containing sodium starch octenylsuccinate and glucose syrup (SSOS + GS) released only 8.79 ± 2.34% of their oil during gastric digestion, compared with 15.91 ± 1.23% for WPI + GS, 22.99 ± 1.45% for WPI + lactose, and 45.67 ± 1.79% for the non-encapsulated algae-oil emulsion. Following transition to intestinal conditions, however, degradation of the SSOS-containing matrix promoted oil release to 67.54 ± 1.78% and FFA release to 54.02 ± 3.72%, compared with only 26.04 ± 1.98% FFA release from the non-encapsulated oil. The latter underwent greater aggregation during gastric digestion, reducing the interfacial area subsequently available for pancreatic lipase. These findings demonstrate that high encapsulation efficiency alone does not predict gastrointestinal performance and that an effective wall should ideally restrict premature gastric release while remaining susceptible to structural reorganization under intestinal conditions.

The same principle applies to multilayer interfacial structures. Ding et al. [128] showed that sequential deposition of alginate and chitosan onto gelatin-stabilized fish-oil droplets substantially increased resistance to gastric coalescence compared with the primary gelatin interface. Nevertheless, the number of deposited layers did not significantly affect the final extent of intestinal digestion, with approximately 80–92% FFA release after 120 min across the multilayer systems. The authors attributed this convergence to the displacement and restructuring of the biopolymer layers by bile salts and pancreatic components after transition to intestinal conditions. Thus, the structural properties responsible for gastric protection do not necessarily persist in the intestine, and the digestive function of an interface depends on its capacity for controlled restructuring rather than on its initial thickness or complexity alone.

### 6.2. Interfacial Remodeling, Lipase Accessibility, and Lipid Digestion

The transition from gastric to intestinal conditions constitutes a critical structural turning point in lipid digestion. The increase in pH modifies biopolymer charge and intermolecular interactions, while bile salts, phospholipids, pancreatic enzymes, and products of lipid hydrolysis compete for the oil–water interface [133,134]. Consequently, the original encapsulating interface may be displaced, partially degraded, penetrated, or reorganized into a mixed interfacial structure. The extent of this remodeling determines whether pancreatic lipase can access the triacylglycerol substrate and whether the resulting lipolysis products can be removed from the interface [133,135].

The susceptibility of the original interface to bile-salt displacement depends strongly on its composition and organization. For example, bile salts completely displaced β-lactoglobulin from the oil–water interface, whereas SPI films were more resistant to displacement [133]. This difference was reflected in lipid digestion: maximum FFA release reached 65.14 ± 0.55% for β-lactoglobulin-stabilized emulsions but only 42.53 ± 0.36% for SPI-stabilized systems. Moreover, the initial rate of lipolysis was strongly related to the specific interfacial area, demonstrating that both interfacial composition and the amount of lipid surface accessible to digestive components contribute to digestion kinetics.

Polysaccharides can further regulate this process by altering the interface’s resistance to bile salts and lipase. Incorporation of peach gum into SPI-stabilized systems, for instance, progressively reduced intestinal FFA release from 82.51% for SPI alone to 70.77% for SPI-PG and 63.60% for the corresponding mixed system [136]. This reduction was associated with the formation of a more viscous and sterically restrictive protein–polysaccharide structure that limited interactions of bile salts and pancreatic lipase with the lipid interface. An even stronger restriction of digestion was obtained by reinforcing a WPI interface with cellulose nanocrystals: maximum FFA release decreased from 45.5% to 16.1%, while the digestion half-time increased from 2.9 to 22.9 min [135]. The dense particle layer resisted bile-salt-mediated interfacial displacement and physically restricted enzyme access. Together, these observations indicate that polysaccharide or particle reinforcement can regulate digestion not only by maintaining droplet stability, but also by controlling the accessibility and displacement of the lipid interface [135,136].

Importantly, resistance to gastric destabilization can also enhance, rather than suppress, intestinal digestion by preserving a finely dispersed lipid phase without preventing subsequent interfacial remodeling. Feng et al. [129] demonstrated this behavior in salmon-oil emulsions stabilized by deamidated wheat gliadin (DWG) and xanthan gum (XG). The addition of 0.3% XG increased adsorbed protein from 58.67% for DWG alone to 73.12% and increased interfacial protein concentration from 0.016 to 0.096 mg/m^2^. This formulation also limited gastric aggregation, thereby preserving a larger lipid-water interfacial area for subsequent pancreatic digestion. Following intestinal interfacial restructuring, the DWG-0.3% XG system exhibited the highest FFA release and EPA/DHA bioaccessibility among the investigated formulations. These results illustrate that a structurally reinforced interface does not inherently reduce lipid digestion; its effect depends on whether the protective architecture remains impermeable in the intestine or instead undergoes controlled displacement and reorganization.

Droplet size and aggregation state provide an additional link between processing and enzyme accessibility. Wang et al. [130] showed that incorporation of DHA-rich fish oil into a heat-denatured WPI Pickering emulsion preserved relatively small lipid droplets (~4.34 µm) during processing and gastrointestinal digestion, whereas the unencapsulated oil formed larger lipid aggregates. After 2 h of intestinal digestion, free DHA accounted for 52% of total DHA in the encapsulated system compared with 40% in the unencapsulated system. Similarly, Jiang et al. [131] reported that high-pressure emulsification of DHA-rich fish oil with dextrin and xanthan gum resulted in greater FFA release (18.18 ± 1.07 mmol/g oil), lipid digestibility (55.11 ± 2.68%), and ex vivo intestinal absorption (16.2%) than bulk or gelatin-capsulated fish oil. Therefore, encapsulation does not necessarily constitute a barrier to digestion; processing strategies that prevent gastric coalescence and maintain a high interfacial area can facilitate subsequent pancreatic lipolysis.

### 6.3. Omega-3 Bioaccessibility and Structure–Function Trade-Offs

Lipid release, lipolysis, and bioaccessibility represent related but distinct stages of gastrointestinal functionality and should not be used interchangeably. Release describes the liberation of the lipid from the encapsulating structure, whereas lipolysis refers to the enzymatic hydrolysis of triacylglycerols, commonly quantified by FFA formation. Bioaccessibility additionally requires the incorporation of released lipid components into the aqueous or mixed-micellar phase, from which they may become available for intestinal uptake [131]. Consequently, extensive oil release does not necessarily indicate efficient omega-3 bioaccessibility, particularly when bile salt availability, micelle formation, lipid molecular structure, or the surrounding food matrix limits solubilization [131,137].

Both the wall-material composition and the processing history of the emulsion can determine how efficiently EPA and DHA enter the bioaccessible fraction. This interaction was evident in spray-dried algae-oil microcapsules produced with either chitosan–maltodextrin or pea protein and homogenized by high-pressure processing or ultrasound [138]. EPA bioaccessibility reached 70.31% and DHA bioaccessibility 63.73% in ultrasound-homogenized pea-protein microcapsules, whereas the corresponding values for high-pressure-homogenized chitosan–maltodextrin particles were 34.42% and 38.21%, respectively. These differences indicate that gastrointestinal performance cannot be attributed to wall-material identity independently of processing, because homogenization determines the initial structure on which subsequent digestive transformations act.

Delivery architecture itself can also be designed to promote rather than merely withstand lipid digestion. In porous silica particles containing fish oil, intestinal fatty-acid production reached 83.5 ± 4.2% and increased to 92.7 ± 5.0% following lecithin incorporation, compared with 64.2 ± 4.9% for commercial fish oil [139]. The silica–lecithin system achieved complete omega-3 bioaccessibility within 60 min. The porous architecture was proposed to promote lipase-substrate interactions while facilitating removal of negatively charged lipolysis products from the reaction interface, thereby limiting product-mediated inhibition. Although this inorganic system differs from protein–polysaccharide matrices, it provides complementary evidence that the delivery system’s architecture can actively facilitate digestion rather than function solely as a passive protective barrier.

Conversely, increasing structural resistance can improve protection while delaying lipid digestion. Bai et al. [140] demonstrated that adding chitosan to SPI-based microcapsules progressively increased the time required to reach the intestinal lipolysis plateau from 15 min for SPI alone to 125 and 145 min for systems containing 0.2% and 0.5% chitosan, respectively. At the same time, increasing chitosan to 0.5% reduced encapsulation efficiency from 79.41 ± 2.72% to 59.75 ± 1.18% and increased surface oil from 4.40 ± 0.44% to 8.10 ± 0.16%. Nevertheless, final lipid digestibility remained 98.2% for the 0.2% chitosan system and 89.1% for the 0.5% system. This illustrates an important distinction between the rate and the extent of digestion: reinforcement of the encapsulating structure may substantially delay lipase access without necessarily preventing eventual lipid hydrolysis.

Furthermore, efficient lipolysis does not guarantee efficient micellization. Final intestinal lipolysis of approximately 72–81% was obtained across emulsions stabilized with pea- or lupin-derived protein ingredients, yet the bioaccessible lipid fraction reached 84–86% for pea systems and only 48–63% for lupin systems [137]. The lower bioaccessibility of the lupin systems was associated with interactions between lupin-derived components and bile salts that impaired mixed-micelle formation without strongly suppressing pancreatic lipolysis. Accordingly, FFA release alone cannot fully characterize gastrointestinal functionality; where possible, evaluation should extend to micellization or direct determination of the bioaccessible omega-3 fraction [137,138].

Taken together, these findings reveal an important structure–function trade-off in the design of omega-3 delivery systems. Increasing matrix density, interfacial thickness, crosslinking, or protein–polysaccharide association can improve resistance to gastric disruption, but excessive structural resistance may also limit bile-salt adsorption, lipase accessibility, and micellarization [132,135,140]. Conversely, interfaces that are readily disrupted can facilitate rapid intestinal lipolysis but may provide insufficient protection during gastric transit [109,134]. The available evidence therefore suggests that the desirable property is not maximal gastrointestinal stability, but stage-specific structural responsiveness: sufficient interfacial and matrix integrity to preserve the dispersed lipid phase during gastric transit, followed by controlled restructuring under intestinal conditions that enables bile-salt-mediated interfacial remodeling, pancreatic lipase access, and incorporation of EPA, DHA, or ALA into mixed micelles [129,134,138]. From a formulation perspective, this shifts the design objective from maximizing individual parameters such as encapsulation efficiency or structural resistance toward balancing protection and digestive accessibility within the complete processing–structure–function pathway, as summarized in Figure 3.

## 7. Methods

### Bibliometric Analysis Using VOSviewer

To position the present review within the broader research landscape, a bibliometric analysis of omega-3 microencapsulation with protein- and polysaccharide-based wall materials was conducted in Scopus. The literature search was performed between August and October 2025 using the search terms “encapsulation”, “omega-3 fatty acids”, “oxidative stability”, “in vitro digestion”, and “microencapsulation methods”. The search was restricted to English-language articles and reviews, resulting in a final dataset of 380 documents. Bibliographic data, including titles, abstracts, and keywords, were exported and analysed using VOSviewer (version 1.6.20). A co-occurrence analysis was performed using author keywords as the unit of analysis and the full-counting method, in which each occurrence of a keyword in a document contributed equally to its total count. A minimum threshold of four occurrences was used to include a keyword in the co-occurrence network, allowing recurrent terms to be retained while maintaining sufficient thematic coverage of the research field. The resulting network was evaluated based on keyword frequency of occurrence, co-occurrence relationships, and thematic clustering. In the network visualization, node size represents the frequency of keyword occurrence, links indicate co-occurrence relationships between keywords, and cluster colors distinguish groups of closely interconnected keywords identified by the VOSviewer clustering algorithm. The resulting author-keyword co-occurrence network is presented in Figure 1.

## 8. Conclusions

This review indicates that the development of effective protein–polysaccharide-based omega-3 delivery systems requires a shift from optimizing individual formulation parameters toward designing the complete processing–structure–function pathway. The available evidence shows that high encapsulation efficiency does not necessarily ensure effective oxidative protection or gastrointestinal performance. Instead, successful formulations should combine sufficient interfacial and matrix integrity to protect omega-3 lipids during processing and storage with controlled structural responsiveness during digestion, allowing lipid release, lipase access, and subsequent bioaccessibility.

From a practical perspective, these findings have direct implications for the formulation of omega-3-fortified foods and functional ingredients. Wall-material combinations and processing conditions should be selected according to the intended application rather than solely to maximize encapsulation efficiency. Systems intended for long-term storage require effective control of surface oil, oxygen transport, and matrix integrity, whereas formulations designed to enhance gastrointestinal delivery must additionally undergo appropriate restructuring under intestinal conditions. Protein–polysaccharide systems are particularly relevant in this context because their interfacial and matrix properties can be tailored through biopolymer composition, processing conditions, and the strength of intermolecular interactions. Such an approach may facilitate the development of omega-3 ingredients with improved storage stability, reduced oxidative deterioration and associated sensory changes, and improved gastrointestinal accessibility of nutritionally relevant omega-3 fatty acids.

Future studies should move toward standardized and application-oriented evaluation of these systems. Conventional oxidation indicators should be complemented by structural descriptors such as surface oil content, oxygen permeability, interfacial characteristics, particle morphology, and matrix physical state, while gastrointestinal studies should clearly distinguish lipid release, lipolysis, and bioaccessibility. Greater use of standardized digestion protocols, realistic food matrices, and storage conditions relevant to commercial products would improve comparability between studies. In addition, in vivo studies are needed to determine whether improvements in gastrointestinal bioaccessibility translate into increased omega-3 bioavailability and to establish their relevance to the recognized nutritional benefits of omega-3 fatty acids.

Overall, integrating processing conditions, protein–polysaccharide interactions, structural characterization, oxidative stability, and gastrointestinal functionality offers a practical route toward more predictive formulation of omega-3 delivery systems. Establishing quantitative relationships among these parameters should reduce reliance on empirical formulation and support the development of stable, food-grade omega-3 ingredients tailored to specific products, storage conditions, and nutritional objectives.

## Figures and Tables

**Figure 1:**
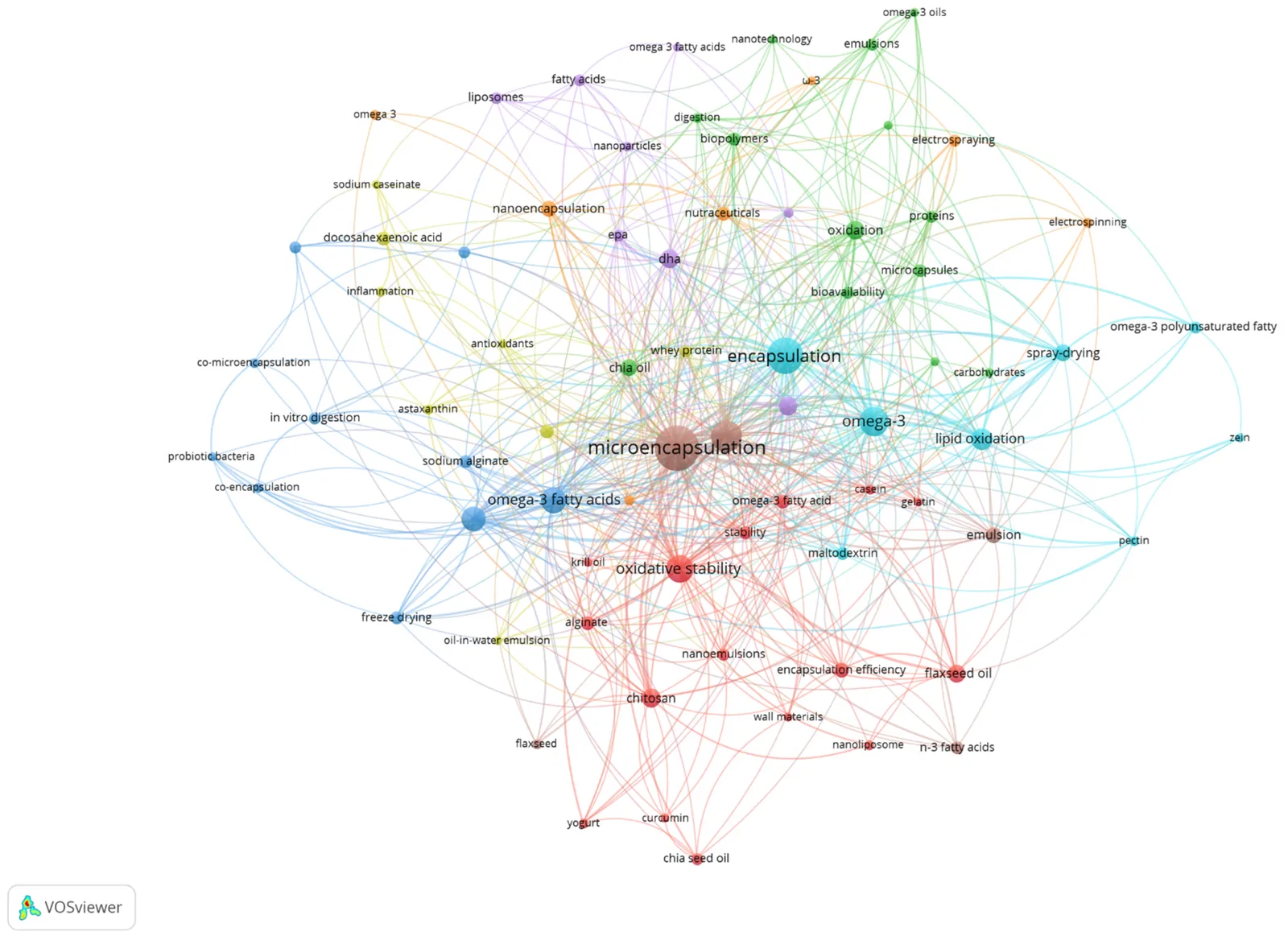
Author-keyword co-occurrence network of research on omega-3 microencapsulation generated using VOSviewer.

**Figure 2:**
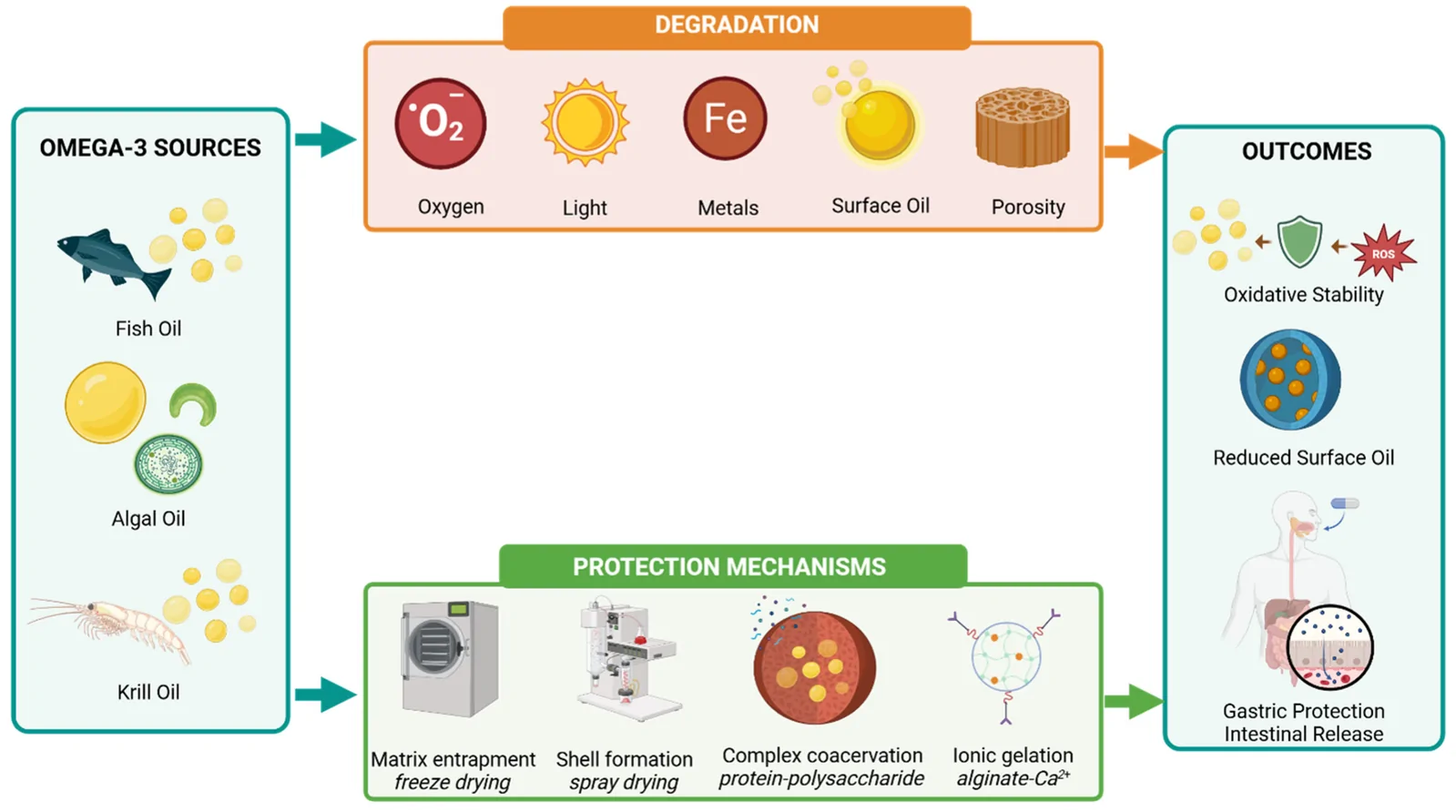
Schematic representation of the major degradation factors affecting omega-3-rich oils and the principal microencapsulation mechanisms used to improve oxidative stability and gastrointestinal functionality.

**Figure 3:**
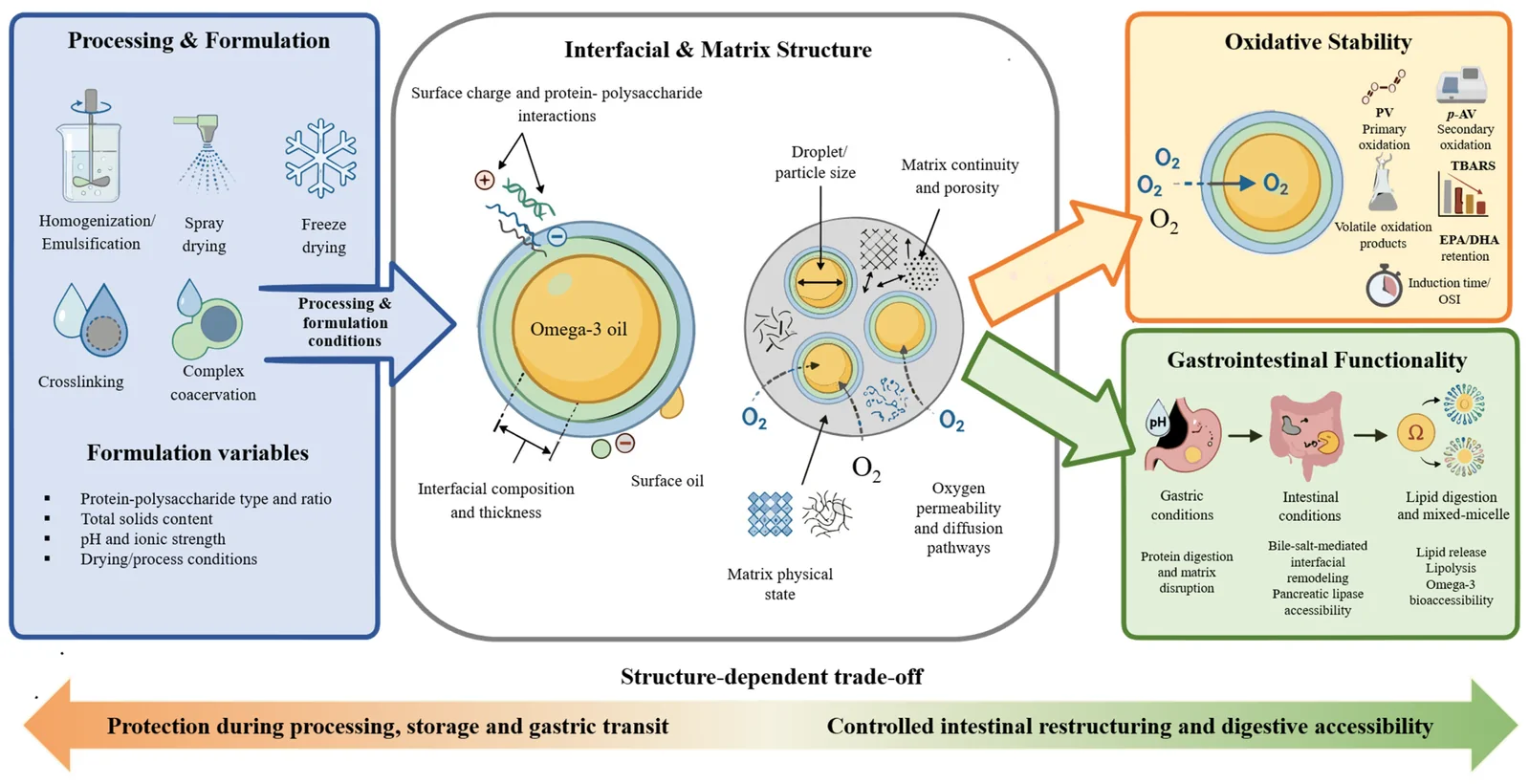
Processing–structure–function framework linking processing and formulation conditions, interfacial and matrix structure, oxidative stability, and gastrointestinal functionality of microencapsulated omega-3 systems.

**Table 1:** Representative wall-material systems used for microencapsulation of omega-3 oils.

Preparation Technique	Wall Materials		Omega-3 Source	Specific Preparation Conditions		Key Outcomes	Reference
	Protein	Polysaccharide/ Carbohydrate		Emulsification Conditions	Powder Formulation Conditions		
Spray drying	Salmon skin gelatin	Gum acacia	Salmon oil	Ultra-Turrax 5000 rpm; 2 min; oil:wall 1:1; FG:GA 1:3	wall:core 1:1; 9% solids; IT/OT 145/55 °C; feed 8–12 mL/min	EE ~100%; PV ~14 meq O_2_/kg oil; PS(D3,2) 11 µm	[61]
Spray drying	Salmon skin gelatin	Maltodextrin; Gum acacia	Salmon oil	Ultra-Turrax 5000 rpm; 2 min; oil:wall 1:1; FG:MD:GA 1:2:1	wall:core 1:1; 9% solids; IT/OT 145/55 °C; feed 8–12 mL/min	EE ~100%; PV ~15 meq O_2_/kg oil; PS(D3,2) 27 µm	
Freeze-drying	BBG	Chitosan	Fish oil	T18 homogenizer 8000 rpm; 5 min	frozen −80 °C overnight; FD 48 h; 20-mesh sieve	EE 75.6%; OLC 17.7%; PV 39.0 ± 3.0 meq O_2_/kg oil (63 °C, 1 h)	[62]
Freeze-drying	BBG	Gum arabic (GA)	Fish oil	T25 homogenizer 8000 rpm; 5 min; oil:BBG 1:1	wall:core 2:1; MD/BBG 0.0; pH 4.0; FD −50 °C 72 h	EE 72.6 ± 4.5%; LC 22.7 ± 0.8%; PVmax 2636 ± 388 meq O_2_/kg oil (60 °C, 1 h)	[63]
Freeze-drying	BBG	GA; Maltodextrin (MD)	Fish oil	T25 homogenizer 8000 rpm; 5 min; oil:BBG 1:1	wall:core 3:1; MD/BBG 1.0; pH 4.0; FD −50 °C 72 h	EE 89.0 ± 1.7%; LC 17.5 ± 1.5%; PVmax 1446 ± 294 meq O_2_/kg oil (60 °C, 3 h)	
Freeze-drying	Fish gelatin (FG)	Sodium alginate (SA)	Fish oil (DHA + EPA ≥ 70%)	Homogenizer 10,000 rpm; 5 min; FG:oil 1:1	FG:SA:oil 4:1:4; pH 4.8; no genipin; FD 48 h	EE ~91 ± 5%; LC ~25.5 ± 2.5%; PVpeak 1318 ± 6 meq O_2_/kg oil	[26]
Uniaxial electrospray-ion gel method	Soy protein isolate	Chitosan	Fish oil (DHA + EPA ≥ 70%)	Homogenizer 8000 rpm; 60 s; CS:SPI 1:1	feed 30 µL/min; needle 0.60 mm; distance 10 cm; 7 kV; gelation in STTP	EE 96.14 ± 1.15%; LC 22.42 ± 0.67%; PV 334 meq O_2_/kg oil (63 °C, 3 h)	[64]
Spray drying	WPC 80	Maltodextrin (DE 14-22)	Linseed oil	Thermomix + HP valve homogenizer (25 & 5 MPa); oil:MD:protein 2:1:1	wall:core 1:1; total solids 20%; disc spray; feed 77 mL/min; IT/OT 125/75 °C	EE 86.98 ± 0.21%; Total oil 49.15 ± 0.95%; OSI 13.08 ± 0.22 h (110 °C; 20 L/h)	[65]
Spray drying	Soy protein	Maltodextrin (DE 14-22)	Linseed oil	Thermomix + HP valve homogenizer (25 & 5 MPa); oil:MD:protein 2:1:1	wall:core 1:1; total solids 20%; disc spray; feed 77 mL/min; IT/OT 125/75 °C	EE 82.62 ± 0.78%; Total oil 49.29 ± 0.30%; OSI 5.05 ± 0.23 h (110 °C; 20 L/h)	
Spray drying	Pea protein	Maltodextrin (DE 14-22)	Linseed oil	Thermomix + HP valve homogenizer (25 & 5 MPa); oil:MD:protein 2:1:1	wall:core 1:1; total solids 20%; disc spray; feed 77 mL/min; IT/OT 125/75 °C	EE 66.68 ± 0.88%; Total oil 45.96 ± 0.58%; OSI 11.73 ± 0.22 h (110 °C; 20 L/h)	
Spray drying	WPC	IRP; Maltodextrin	DHA-rich algal oil	Mech. stir 1500 rpm 2 h; homogenizer 7 min; 25% oil load	30% solids; IRP:MD:WPC 1:3:1; IT/OT 180/80 °C; feed 25 mL/min	EE 85.20 ± 6.03%; PV increased from ~94.6 to ~173.4 meq O_2_/kg oil over 12 days at 60 °C; PS 631.60 ± 23.19 nm	[66]
Freeze-drying	PSG (80 mg/mL)	SHMP; SSOS	Fish oil	Homogenization 8000 rpm; 5 min; 8 g oil/100 mL water	SHMP:gelatin 1:15; pH 4.7; 50 °C; 8 g SSOS; −50 °C 48 h	EE 98.2%; LC 26.4%; PVmax ranged from 153 ± 5 to 168 ± 3 meq O_2_/kg oil (3 h)	[67]
Spray drying	Whey protein	Gum arabic	Chia oil	Magnetic stirring 100 rpm 12 h RT; oil:wall 1:5; WP:GA 1:1	wall:core 5:1; IT 150 °C; OT 82 ± 3 °C; feed 5.5 mL/min; nozzle 0.7 mm	EE 65.81 ± 0.49%; Payload 16.6 g/100 g; PV increased from 1.17 ± 0.28 to 20.83 ± 0.76 meq O_2_/kg oil over 6 months	[48]
Freeze-drying	Whey protein	Gum arabic	Chia oil	Magnetic stirring 100 rpm 12 h RT; oil:wall 1:5; WP:GA 1:1	freezing −80 °C; vacuum 0.1 mbar; 48 h; shelf RT	EE 56.60 ± 0.17%; Payload 16.6 g/100 g; PV increased from 1.17 ± 0.28 to 18.17 ± 1.04 meq O_2_/kg oil over 6 months	
Spray drying	WPC	Maltodextrin	Cod liver oil	Thermomix 9000 rpm 120 s 40 °C; valve homogenizer 240/40 bar; 10% oil	wall:core 1:1; disc 110 mm (6400 rpm); IT/OT 130/90 °C; feed 77 mL/min	EE 65.70 ± 0.52%; Total oil 40.66 ± 0.39%; OSI 12.67 ± 0.08 h (110 °C)	[68]
Spray drying	Soy protein	Maltodextrin	Cod liver oil	Thermomix 9000 rpm 120 s 40 °C; valve homogenizer 240/40 bar; 10% oil	wall:core 1:1; disc 110 mm (6400 rpm); IT/OT 130/90 °C; feed 77 mL/min	EE 63.66 ± 1.87%; Total oil 38.62 ± 0.46%; OSI 6.38 ± 0.07 h (110 °C)	
Spray drying	Lentil protein isolate (LPI)	ι-Carrageenan; Maltodextrin	Flaxseed oil	Polytron 15,000 rpm; 2 × 5 min; oil load 7.5%	wall:core 2.33:1; IT/OT 135 ± 1/95 ± 2 °C; air 473 L/h; feed 3 mL/min	EE 83.51 ± 2.16%; PV 6.7 meq O_2_/kg oil; TBARS ~0.7 mg MDA/g	[50]
Spray drying	WPC hydrolysate	Glucose syrup (DE38)	Refined fish oil	Ultraturrax 15,000 rpm 2 min; HPH 450/75 bar; 3 passes; 5% oil	Total solids 39%; IT/OT 180/90 °C; Air flow 25 Nm^3^/h	EE >87%; PS 9.37 ± 5.20 to 10.54 ± 5.26 µm	[69]

PV values originally reported in mmol/kg oil were converted to meq O_2_/kg oil using 1 mmol peroxide/kg = 2 meq O_2_/kg. Values originally reported as g I_2_/100 g oil were converted to meq O_2_/kg oil using the iodine equivalent specified in the original method. Abbreviations: BBG, bovine bone gelatin; CS, chitosan; DHA, docosahexaenoic acid; EPA, eicosapentaenoic acid; EE, encapsulation efficiency; FD, freeze-drying; FG, fish gelatin; GA, gum arabic (gum acacia); HPH, high-pressure homogenization; HP, high-pressure; IRP, infant rice powder; IT, inlet temperature; OT, outlet temperature; LC, loading capacity; MD, maltodextrin; OLC, oil loading capacity; OSI, oxidative stability index;PSG/FG (as used in the source study), type/source of gelatin; PS, particle size; PV, peroxide value; RT, room temperature; SA, sodium alginate; SHMP, sodium hexametaphosphate; SPI, soy protein isolate; SSOS, starch sodium octenyl succinate (OSA-modified starch); STTP, sodium tripolyphosphate; TBARS, thiobarbituric acid reactive substances; TBA, thiobarbituric acid assay; WPC, whey protein concentrate.

## Data Availability

No new data were created or analysed in this study. Data sharing is not applicable to this article.

## Abbreviations

The following abbreviations are used in this manuscript:

ALA	α-Linolenic acid
BBG	Bovine bone gelatin
BSG	Bovine skin gelatin
CS	Chitosan
DE	Dextrose equivalent
DHA	Docosahexaenoic acid
EE	Encapsulation efficiency
EPA	Eicosapentaenoic acid
ESR	Electron spin resonance
FD	Freeze-drying
FG	Fish gelatin
GA	Gum arabic (gum acacia)
HP	High pressure
HPH	High-pressure homogenization
IRP	Infant rice powder
IT	Inlet temperature
LC	Loading capacity
LO	Linseed oil
LPI	Lentil protein isolate
MD	Maltodextrin
MDA	Malondialdehyde
O/W	Oil-in-water
OLC	Oil loading capacity
OSI	Oxidative stability index
OT	Outlet temperature
Pec	Pectin
PM	Pea protein
PS	Particle size
PSG	Porcine skin gelatin
PUFAs	Polyunsaturated fatty acids
PV	Peroxide value
RT	Room temperature
SA	Sodium alginate
SHMP	Sodium hexametaphosphate
SIO	Sacha inchi oil
SPI	Soy protein isolate
SSOS	Starch sodium octenyl succinate
STTP	Sodium tripolyphosphate
TBA	Thiobarbituric acid assay
TBARS	Thiobarbituric acid reactive substances
WPC	Whey protein concentrate
WP	Whey protein
WPI	Whey protein isolate
p-AV	*p*-Anisidine value
FFA	Free fatty acids
TOTOX	Total oxidation value
DWG	Deamidated wheat gliadin
XG	Xanthan gum
SG	Spray granulation
SD	Spray drying
VSD	Vacuum spray drying
